# Limited associations between MHC diversity and reproductive success in a bird species with biparental care

**DOI:** 10.1002/ece3.10950

**Published:** 2024-02-20

**Authors:** Diana Ferreira, Luis M. San‐Jose, Alexandre Roulin, Arnaud Gaigher, Luca Fumagalli

**Affiliations:** ^1^ Laboratory for Conservation Biology, Department of Ecology and Evolution, Biophore University of Lausanne Lausanne Switzerland; ^2^ Laboratoire Évolution and Diversité Biologique, UMR 5174, CNRS Université Toulouse III Paul Sabatier, IRD Toulouse France; ^3^ Department of Ecology and Evolution, Biophore University of Lausanne Lausanne Switzerland; ^4^ CIBIO‐InBIO, Research Center in Biodiversity and Genetic Resources University of Porto Vairão Portugal; ^5^ Research Unit for Evolutionary Immunogenomics, Department of Biology University of Hamburg Hamburg Germany; ^6^ Swiss Human Institute of Forensic Taphonomy, University Centre of Legal Medicine Lausanne‐Geneva Lausanne University Hospital and University of Lausanne Lausanne Switzerland

**Keywords:** birds, functional divergence, major histocompatibility complex, reproductive success, supertype

## Abstract

The selective pressure from pathogens on individuals can have direct consequences on reproduction. Genes from the major histocompatibility complex (MHC) are central to the vertebrate adaptive immune system and pathogen resistance. In species with biparental care, each sex has distinct reproductive roles and levels of investment, and due to a trade‐off with immunity, one can expect different selective regimes acting upon the MHC of each parent. Here, we addressed whether couples combine each other's variation at MHC loci to increase their breeding success. Specifically, we used a 23‐year dataset from a barn owl population (*Tyto alba*) to understand how MHC class Iα and IIβ functional divergence and supertypes of each parent were associated with clutch size and fledging success. We did not detect associations between MHC diversity and supertypes with the clutch size or with the fledging success. In addition, to understand the relative contribution from the MHC of the genetic parents and the social parents, we analyzed the fledging success using only a cross‐fostered dataset. We found several associations of weak‐to‐moderate effect sizes between the father's MHC and fledging success: (i) lower MHC‐Iα divergence in the genetic father increases fledging success, which might improve paternal care during incubation, and (ii) one and two MHC‐IIβ DAB2 supertypes in the social father decrease and increase, respectively, fledging success, which may affect the paternal care after hatching. Furthermore, fledging success increased when both parents did not carry MHC‐IIβ DAB1 supertype 2, which could suggest conditional effects of this supertype. Although our study relied on a substantial dataset, we showed that the associations between MHC diversity and reproductive success remain scarce and of complex interpretation in the barn owl. Moreover, our results highlighted the need to incorporate more than one proxy of reproductive success and several MHC classes to capture more complex associations.

## Graphical abstract

Using a long‐term monitoring dataset of a wild barn owl population, we investigated how MHC diversity and supertypes are associated with clutch size and fledging success. We found a weak‐to‐moderate effect of the MHC diversity and specific supertypes of the fathers on the fledging success but not on the clutch size. We showed that the associations between MHC diversity and reproductive success remain scarce and of complex interpretation in the barn owl.

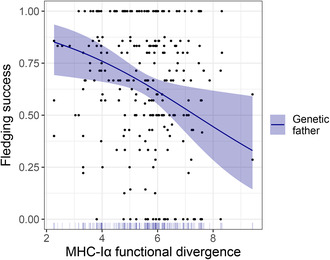

## 1 INTRODUCTION

The major histocompatibility complex (MHC) multigene family plays a crucial role in the vertebrate adaptive immune response and ultimately pathogen resistance (Radwan et al., 2020). Classical MHC loci encode for cell surface glycoproteins that bind antigen‐peptides to be recognized by T cells and trigger an immune response where MHC class I (MHC‐I) molecules recognize intracellular pathogens (e.g., viruses), whereas class II (MHC‐II) recognize extracellular pathogens (e.g., bacteria, parasites) (Hughes & Yeager, 1998). Because both classical MHC‐I and MHC‐II genes have evolved an extraordinarily high level of functional diversity (from basal jawed vertebrates to mammals; Gaigher et al., 2023; Kaufman, 2018), they became the target of intense research to understand the selective forces involved in maintaining such variation (e.g., Piertney & Oliver, 2006; Radwan et al., 2020; Spurgin & Richardson, 2010). The link between fitness and genetic variation in the MHC genes is often considered from the prism of how MHC variants differentially protect against pathogens and generate differences in survival across individuals (e.g., Bateson et al., 2016; Radwan et al., 2020; Spurgin & Richardson, 2010). However, it was also observed that MHC diversity was not correlated with infection susceptibility or intensity in species with depleted MHC diversity (Gutierrez‐Espeleta et al., 2001; Radwan et al., 2010). Infection and reproductive effort represent a central issue in life‐history trade‐offs (Gustafsson et al., 1994; Knowles et al., 2010); consequently, it is expected that variation at the MHC could ultimately underpin differences in the reproductive success among individuals.

Reproductive success is a relevant fitness trait to understand how MHC mediates adaptation because the selection imposed by pathogens can have direct consequences on individuals' reproductive investment (a proxy that integrates both health and vigor; Apanius et al., 1997; Bonneaud et al., 2003; Eizaguirre et al., 2009; Marzal et al., 2005). The MHC composition can influence reproductive success in two non‐exclusive ways (Kalbe et al., 2009; Zelano & Edwards, 2002). First, a given MHC composition can increase the quality of the parent and consequently the quality of the parental care (e.g., amassing territory and resources; Roved et al., 2018). Second, parents can transmit advantageous MHC alleles that increase the quality and survival of their offspring (Brouwer et al., 2010). MHC might influence reproductive success differently in males and females (Hoover et al., 2018; Jäger et al., 2007; Kalbe et al., 2009; Roved et al., 2018). Sexes optimize reproductive success by different means, given the distinct roles of males and females during reproduction and the differences in the level or the timing of reproductive investment (Jennions & Fromhage, 2017; Liker et al., 2015). In addition, there are well‐recognized differences between sexes in immunity (Klein & Flanagan, 2016; Love et al., 2005; Valdebenito et al., 2021) and even in the way MHC regulates the immune response of males and females (Pineaux et al., 2020; Roved et al., 2017).

Several studies have shown an association between MHC diversity and reproductive success (e.g., Hoover et al., 2018; Huang et al., 2022; Kalbe et al., 2009; Lenz et al., 2013; Pikus et al., 2022; Roved et al., 2018; Sepil, Lachish, & Sheldon, 2013b). For instance, specific MHC‐I supertypes explained higher reproductive success in great tits, *Parus major* (Sepil, Lachish, & Sheldon, 2013b), whereas in the Galápagos sea lions, *Zalophus wollebaeki*, MHC‐II divergence was involved instead (Lenz et al., 2013). However, these studies usually targeted only one component of the MHC adaptive immunity, either MHC‐I or MHC‐II (but see Pikus et al., 2022). Given that genes of different MHC classes are responsible for the detection of pathogens of different natures and, thus, under different selective pressures (Minias et al., 2018), a multi‐locus approach is needed to better understand the extent of MHC‐fitness associations (Gaigher et al., 2019; Kamiya et al., 2014).

Additionally, it is still poorly known how the combination of variation at MHC loci of male and female mates influences their reproductive success, which contrasts with the number of studies conducted to understand how MHC is involved in mate choice (e.g., Kamiya et al., 2014; Stiver & Alonzo, 2009; Zelano & Edwards, 2002). One could predict that a male or a female modulates its investment in reproduction based on the MHC quality of its mate (Sheldon, 2000; Wedell & Karlsson, 2003; Zelano & Edwards, 2002). To the best of our knowledge, the only study that explores this question was conducted on blue peafowl, *Pavo cristatus* (Hale et al., 2009). The authors observed that females laid more and heavier eggs when mated with males with higher MHC‐I diversity, suggesting that females assess the MHC of the male and invest accordingly. However, this study relied on (i) a specific reproductive system (lek mating), where males do not provide parental care, and (ii) a captive breeding experiment. Consequently, whether the parents' MHC genotypes have similar or rather different relative contributions to the successful breeding, whether maternal and paternal effects are additive, or whether the contribution of one parent's genotype is dependent on the MHC diversity of its partner remains to be addressed in wild populations.

Here, we investigate how father and mother MHC functional divergence and supertypes affect their reproductive success using a multi‐locus approach, studying both MHC class I and II genes of an outbred wild barn owl, *Tyto alba*, population in Switzerland. In this species, the MHC is characterized at MHC‐Iα and MHC‐IIβ, and both have a rather simple organization (two genes at each class, Burri, Niculita‐Hirzel, et al., 2008b; Gaigher et al., 2018, Gaigher et al., 2016), which is advantageous for detecting precise relationships between MHC diversity and fitness components. In addition, the barn owl is socially monogamous and both sexes have marked parental roles, and therefore represents a good model species to investigate whether couples combine their MHC genotypes to improve their reproductive success (Roulin, 2020). Laying eggs is energetically demanding for the female, and she is responsible for incubation and brooding the young until 2 weeks after hatching (Roulin, 2020). After this period, the female leaves the nest and may help the male feed the chicks at approximately half his rate or start a new clutch with another male (Durant et al., 2004; Roulin, 1999, 2002a). The male is responsible for feeding the female while she is laying and incubating, and feeding the chicks until they fledge; hence, his presence is essential for the conclusion of the brood (i.e., that all chicks fledge, Almasi et al., 2008). When the couple performs poorly in a breeding season, they usually divorce, acquire a higher quality or more compatible mate, and increase reproductive success in the next year, which suggests that successful reproduction depends on the combination of partners (Dreiss & Roulin, 2014).

In the present study, we used 23 years of breeding records of a barn owl population to understand how the couples' MHC divergence and supertype combinations influence their clutch size and fledging success. Given that a couple's reproductive success is a co‐joint effort, we hypothesize that the influence of one parent MHC is dependent on the MHC of its partner. Alternatively, both parents' MHC can influence their reproductive success independently of the partners' MHC through direct or indirect links based on the relative roles of each parent. Using a subset of the data subjected to an experimental cross‐fostering protocol (e.g., Roulin & Dijkstra, 2003), an additional objective was to understand whether the MHC of genetic parents shows different relative contributions from the social parents on the fledging success. If the MHC functional divergence and/or supertypes of the genetic parents significantly explain variation in the fledging success, then it would suggest that MHC acts via genetic inheritance toward the offspring (Brouwer et al., 2010) or via parental care before cross‐fostering (e.g., attentiveness during incubation). Alternatively, if the MHC of the social parents explains the fledging success, the MHC could act via parental care, most likely reflecting the fitness of the social parents (Roved et al., 2018).

## 2 MATERIALS AND METHODS

### 2.1 Study species and breeding monitoring

The barn owl is a medium‐sized nocturnal bird of prey that mainly lives in open agricultural landscapes and hunts small mammals. Barn owls are socially monogamous and produce up to two annual broods from February to October (Béziers & Roulin, 2016). Since 1990, 350 next boxes have been installed on farm buildings between lakes Léman and Neuchâtel in western Switzerland (46°490 N, 06°560 E). The clutch size varies from 2 to 12 (mean: 6.23 ± 1.64 SD), and the brood size at fledging varies from 0 to 10 (mean: 3.62 ± 2.01 SD). Each egg is laid every 2–3 days, and incubation starts with the first egg laid, resulting in asynchronous hatching and pronounced age hierarchy among the offspring (Roulin, 2002b). Nestlings fledge at around 55 days of age.

All nest boxes were visited monthly from March to September. When a clutch was found, embryo development was assessed with egg candling, and ca. 25 days after the first egg laid, the female was captured and the final number of eggs laid counted, that is, clutch size. The male was captured when feeding the nestlings. The clutch was visited again when the oldest chick was 55 days old and re‐visited when the youngest chicks also reached 55 days to determine the number of fledglings. The number of fledglings represents the offspring's survival up to fledging, and it serves as a good proxy of parental care since this species is altricial (i.e., dependent on parental provisioning). For each brood, we calculated the fledging success, corresponding to the proportion of chicks that fledge given the clutch size. In this population, clutch size and fledging success show individual variability and temporal variation (i.e., condition dependency of individual reproductive fitness; Altwegg et al., 2007), so they are appropriate to test for associations with genes underlying fitness traits at different stages of the reproduction. We avoided exploring whether MHC diversity explained lifetime reproductive success (total number of offspring produced over a lifetime; for example, Sepil, Lachish, & Sheldon, 2013b), as this metric is not suitable to investigate the effect of each parent's MHC on brood success due to a high divorce rate and mate change due to the death of one partner in this barn owl population (ca. up to 50% between first and second annual breeding attempts or between successive years; Béziers & Roulin, 2016, Dreiss & Roulin, 2014), which would result in low sample sizes for each couple.

We used cross‐fostering methods to disentangle the effect of origin‐related factors (e.g., genetics) from the effects of environmental factors (e.g., rearing) to understand phenotypic variation and responses to selection (Winney et al., 2015). We matched the pairs of nests by hatching date and clutch size to maintain the same hierarchical structure (Winney et al., 2015). When nests only differed in no more than one egg/nestling, we swapped all eggs/chicks between nests (i.e., full cross‐fostering). In case of uneven clutch sizes, we only swapped a certain number of eggs (i.e., partial cross‐fostering), and the age hierarchy of eggs/chicks was kept intact (Winney et al., 2015). All parentage was confirmed by genotyping all chicks at 10 microsatellite markers (multiplex 3 and 4; Burri, Antoniazza, et al., 2008a). This study gathered 1190 individuals which produced 1079 clutches between 1994 and 2017, and in which 370 were not cross‐fostered, 329 were fully cross‐fostered, and 380 were partially cross‐fostered. Due to field limitations, 6% of the eggs swapped in the full cross‐fostering experiment had already hatched. Even though we tried to swap shortly after hatching, some chicks have experienced parental care in the nest of origin for a very limited amount of time. Blood samples were taken from the brachial vein of the captured adults and stored either in EDTA or immediately centrifuged, and the whole blood was kept at −4°C until stored in the laboratory at −80°C.

### 2.2 MHC sequencing and genotyping

A total of 1190 adults were genotyped, where 989 were genotyped in this study, while the remaining ones came from previous studies (see Gaigher et al., 2018, 2019). DNA was extracted from blood with the DNeasy Blood and Tissue kit (Qiagen) following the manufacturer's protocol. We investigated exon‐3 from the two loci of the MHC‐Iα gene and exon‐2 from the two loci of the MHC‐IIβ gene (hereafter MHC‐IIβ DAB1 and MHC‐IIβ DAB2). These regions encode for the highly polymorphic peptide‐binding region responsible for antigen recognition and are the most studied regions in avian MHC research (Minias et al., 2018). Our PCR protocols co‐amplified both loci of MHC‐Iα, while the two loci of MHC‐IIβ were amplified independently, using an individual dual barcode strategy designed for high‐throughput sequencing (see Section S1). PCR products were checked on a 1.5% agarose gel. Barcoded samples were pooled into two libraries according to their barcode combination and equimolar concentrations and sequenced with a 250 bp paired‐end MiSeq protocol (Illumina) in a single run by Fasteris (Geneva, Switzerland). We included 120 replicate samples from independent PCR to evaluate sequencing repeatability. All details for MHC primer development, PCR amplification, and high‐throughput sequencing are referred in Gaigher et al. (2016) for MHC‐Iα, and in Burri, Niculita‐Hirzel, et al. (2008b) and Gaigher et al. (2018) for MHC‐IIβ. The filtering procedure applied in this study to remove low‐quality sequences and rare artefactual variants is similar to the one described in Gaigher et al. (2016, 2018) and was performed with OBITOOLS (Boyer et al., 2016).

The genotyping protocol was based on the “Degree of Change” (Lighten et al., 2014) and the “Threshold method” (Galan et al., 2010) that have previously been confirmed to accurately provide MHC genotypes in this species based on allelic segregation within pedigrees (see Gaigher et al., 2016 and Section S1). Both methods fit particularly well with our current datasets, which are characterized by only two duplicated genes, high coverage, and a low number of artifacts. Finally, outputs yielded by these methods were manually screened in ClustalW within MegaX (Kumar et al., 2018) to remove potential chimeric variants and non‐functional variants (frameshift, stop codons), but none were detected. Our genotyping was considered reliable due to the congruent genotypes between the two methods, the replicates (98% reproducibility), and the pattern of allelic segregation from related individuals in our dataset.

### 2.3 MHC diversity estimates

We estimated individual MHC diversity for each locus using two different allelic distances based on the entire exon: (i) the amino acid p‐distance between the alleles, calculated in MegaX (Kumar et al., 2018), and (ii) the amino acid functional divergence between the alleles, calculated with Grantham's distance using aminoacids' physicochemical properties (perl script from Pierini & Lenz, 2018; Grantham, 1974). Due to the co‐amplification of the two loci of MHC‐Iα, these distances were calculated using the total average distances between all alleles (maximum of four alleles expected). Although copy number variation has been previously suggested for MHC‐Iα in the barn owl (Gaigher et al., 2016, 2018), we used the total number of different MHC‐Iα alleles per individual to avoid bias. As p‐distances and functional divergences showed strong correlations (*r* > 0.85), we only kept the functional divergence as an explanatory MHC variable for further statistical analysis. This method of quantifying MHC diversity has been gaining traction over the last few years as a more meaningful way of measuring diversity in MHC loci (e.g., Arora et al., 2020; Grieves et al., 2019; Leclaire et al., 2019; Lenz et al., 2013; Pierini & Lenz, 2018; Pineaux et al., 2020).

### 2.4 Supertype clustering

Since not all substitutions among MHC alleles translate into functionally significant differences in the peptide‐binding process, alleles were clustered into supertypes. Clustering was based on the overlap of physicochemical properties of codon sites affected by positive selection so that similar peptide‐binding motifs would be clustered into the same supertype (Doytchinova & Flower, 2005; Trachtenberg et al., 2003). First, for each gene, we inferred the amino acids showing evidence of positive selection with CodeML implemented in PAML v4.8 (Yang, 2007; see Section S2). Second, we constructed a matrix with each positively selected amino acid (as rows) characterized by five physicochemical metrics (as columns; Doytchinova & Flower, 2005). Third, with this matrix, we selected the optimal number of clusters with two K‐means algorithms: Bayesian Information Criterion (adegent R package, Jombart, 2008) and the “*silhouette*” method (factoextra R package, Kassambara & Mundt, 2020) for MHC‐Iα and MHC‐IIβ, respectively. Finally, alleles were clustered by discriminant analysis of principal components with the adegent R package (Jombart, 2008). This procedure is now widely used for its biological relevance and statistical practicality (e.g., Lillie et al., 2015; Migalska et al., 2019; Phillips et al., 2018; Schwensow et al., 2008; Sepil, Lachish, & Sheldon, 2013b; Smallbone et al., 2020). Even though grouping alleles into supertypes allows the inclusion of less frequent alleles (i.e., rare) without hampering statistical power, rarer, highly divergent alleles may not cluster with any other allele and therefore not be included in further analysis. Thus, to prevent a lack of statistical power, we only analyzed the supertypes with a frequency above 0.10. The frequency of each supertype in the population was calculated as the number of individuals carrying it divided by the total number of individuals genotyped for each gene.

### 2.5 Statistical analysis

To test if individuals combine and potentially adjust their reproductive effort driven by their partners' MHC composition, we performed generalized linear mixed models using clutch size (Poisson response with a *log‐link*) and fledging success (Binomial response with a *logit‐link* and weighted with the clutch size) as dependent variables. All models were run separately for each MHC variable (functional divergence and supertypes) and gene since they represent different immune functions and are under different selection pressures (Gaigher et al., 2018; Minias et al., 2018). This also helped to avoid over‐parametrization of the models.

First, using the entirety of the dataset, we built a set of full models including the following fixed factors: the interaction of mother and father MHC functional divergences (i.e., testing for conditional associations), the linear effects of mother and father functional divergences and respective quadratic terms (i.e., testing for non‐linear relationships as previously observed in other systems, e.g., Kalbe et al., 2009), the age of the mother and the father (in years; Altwegg et al., 2007), and the laying date (in Julian days) and its quadratic term to account for the effect of seasonality (Chausson et al., 2014). Then, we extended the analysis with the dataset containing only the cross‐fostered clutches to investigate the relative contribution of the MHC of the genetic parents (i.e., genetic effects) versus the MHC of the social parents (i.e., rearing effects) to the fledging success. The models followed the described rationale but included the MHC variables of both genetic and social parents in the same models. For all models, individual identities and year of breeding were set as random factors to control for repeated individual observations and annual environmental heterogeneity, respectively. The assumptions of the full models were verified, and no correction was needed. There was no collinearity between the variables tested (VIFs < 2).

Following Grueber et al. (2011), we used an information‐theoretic approach to simplify the full models and obtain the simplest model maximizing the variance explained (i.e., multimodel inference; Burnham et al., 2011; Garamszegi et al., 2009; Symonds & Moussalli, 2011). First, we standardized the full model to be able to directly compare the effects between predictors (Gelman, 2008; Grueber et al., 2011). Second, we used the full standardized models to derive all possible sub‐models with MuMIn's R package function *dredge*. For a correct statistical interpretation of the model estimates, we used the *subset* argument to ensure that models where interactions but not the main effects of the interactions were not considered. The covariates parental age and laying date were added to all models (including the null models) using the argument *fixed* (we do not show the results concerning these variables as their effects are out of the scope of this study). Third, we selected the sub‐models by ranking them according to their AICc (Akaike information criterion corrected for small sample sizes) with the argument *rank*. For each model selected, Akaike weight (*w*) is calculated to assess the probability of each model being the best model given the data and the other models in the set of models. Lastly, models with a ΔAICc ≤ 2 from the model with the lowest AICc score were retained in the set of best models, as they can be considered as equally well supported, and were fully averaged with the *model.avg* function to get averaged standardized estimates (β) and statistical significance based on 95% confidence intervals while considering uncertainty in model selection. A variable was considered to significantly explain the clutch size and fledging success when the 95% CI for the estimates excluded zero.

For the set of models testing the effect of the MHC supertype, we fitted one model for each supertype to avoid overfitting due to the high number of supertypes present in this population with a frequency higher than 0.10 (Table S6). Contrary to the multimodel inference taken above, given the binary nature of the supertype data, a traditional null testing hypothesis approach was employed instead. The models included the presence/absence of that supertype on each parent, coded as 0/1 respectively, along with their interaction, the ages, and the laying date and its quadratic term. For the extended analysis with cross‐fostering data, we also included the variables concerning the social parents. Individual identity and year were added as random factors. We adjusted the significance level of multiple tests using the Benjamini‐Hochberg procedure (Yoav & Yosef, 1995). Upon significant interactions, we calculated contrasts among couples regarding the presence/absence of specific supertypes with post‐hoc Tukey pairwise comparisons of estimated marginal means.

For our first investigation, we used 935, 895, and 893 to analyze the effects of MHC‐Iα, MHC‐IIβ DAB1, and MHC‐IIβ DAB2 (Tables S1 and S2), respectively. The sample size for the second investigation based on fully cross‐fostered clutches included 243, 214, and 213 clutches for MHC‐Iα, MHC‐IIβ DAB1, and MHC‐IIβ DAB2 (Tables S3 and S4), respectively. All analyses were performed in R (RStudio Team, 2020), using packages LME4 (Bates et al., 2015) to build all models, arm (Gelman et al., 2020) for model standardization, MuMIn (Bartón, 2020) for model construction, selection, and averaging, and emmeans (Russell et al., 2021) for computing estimated marginal means and post‐hoc pairwise comparisons. Plotting of the coefficients and estimated marginal means was performed with ggplot2 (Wickham et al., 2022) and cowplot (Wilke, 2022).

In our preliminary analyses, neutral diversity (standardized observed heterozygosity based on 10 microsatellite markers, Burri, Antoniazza, et al., 2008a) was included in all models to control for genome‐wide diversity. However, as neutral diversity never attained significance in our models, this predictor was not included in our final set of models.

## 3 RESULTS

### 3.1 Genotyping, MHC divergence and supertypes

We found 96, 31, and 22 distinct alleles for MHC‐Iα, MHC‐IIβ DAB1, and DAB2, respectively (genotyping success was high, ≥96% for all loci). Compared to previous works on this population (Gaigher et al., 2016, 2018), we found 13, 2, and 3 new alleles for MHC‐Iα, MHC‐IIβ DAB1, and DAB2, respectively (Tables S3 and S4; accession numbers provided in Tables S3 and S4). By clustering alleles with similar amino acid properties at residues evolving under positive selection, we obtained 9, 14, and 10 distinct supertypes for MHC‐Iα, MHC‐IIβ DAB1, and MHC‐IIβ DAB2, respectively (Tables S5 and S6, Figures S1–S3). A summary of MHC functional divergence and supertypes present in the population is shown in Table 1.

**TABLE 1:** Summary of MHC diversity and supertypes.

	Nb alleles	Functional divergence (SD)	Sites + selected	Nb supertypes	Mean nb supertypes (SD)
MHC‐Iα	98	5.402 (1.402)	8	9	3.064 (0.694)
Female	92	5.310 (1.414)		9	3.041 (0.688)
Male	87	5.521 (1.377)		9	3.093 (0.721)
MHC‐IIβ DAB1	31	8.229 (4.473)	15	14	1.815 (0.388)
Female	27	8.235 (4.447)		14	1.804 (0.397)
Male	28	8.221 (4.505)		12	1.824 (0.381)
MHC‐IIβ DAB2	22	3.528 (2.669)	10	10	1.635 (0.481)
Female	21	3.494 (2.646)		10	1.634 (0.482)
Male	20	3.573 (2.694)		10	1.637 (0.481)

*Note*: Functional divergence: amino acid functional divergence between alleles; Sites + selected: number of codons under positive selection and used to cluster alleles into supertypes; Mean nb supertypes: mean individual number of supertypes. Standard deviations, SD, are in brackets.

### 3.2 Association of MHC divergence and supertypes with clutch size

**FIGURE 1:**
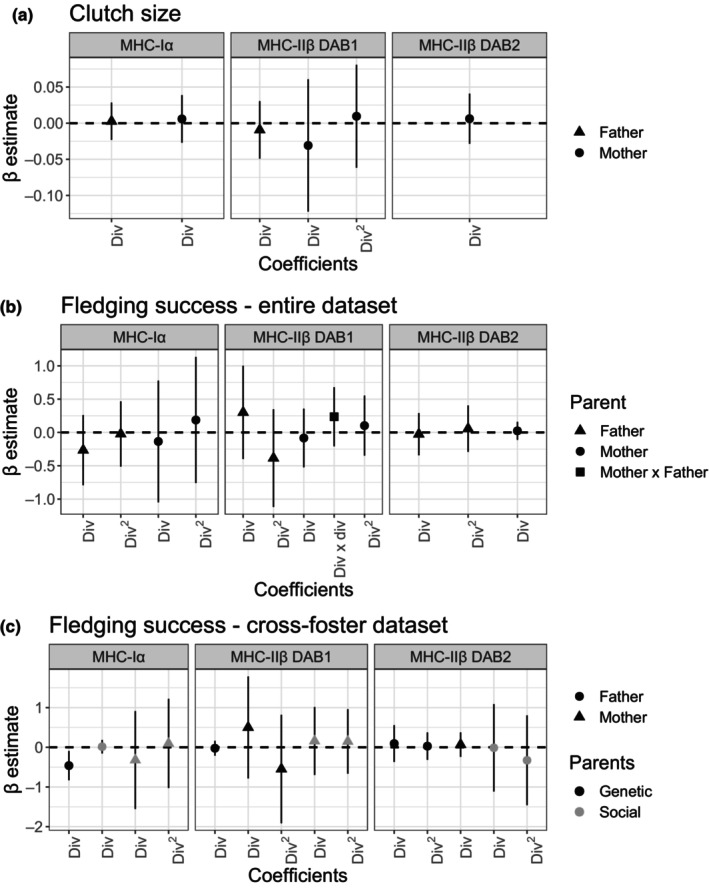
Model‐standardized (β) averaged estimates of MHC functional divergence coefficients of genetic (lighter) and social parents (darker) on clutch size (a), fledging success with the entire dataset (b), and fledging success with the cross‐fostering dataset (c). Each panel shows the effect size of each MHC functional divergence (dots and triangles) included in the full‐averaged model and their 95% confidence intervals. Coefficients in which their 95% CI does not cross the dashed line are considered significant. Covariates are omitted for simplification (see Tables S8, S11, and S15 for entire model information). Values presented are in the scale of the response variable: *log‐link* for clutch size and *logit‐link* for fledging success. Div, MHC functional divergence; Div^2^, quadratic term of the MHC functional divergence; Div × div, interaction of fathers' and mothers' MHC functional divergences.

Our results do not support an association between clutch size and mothers' or the fathers' functional divergence at any MHC loci. The models with ΔAICc ≤ 2 did not include the interaction between the mothers' and fathers' functional divergence for none of the analyzed loci, which suggests that the combination of MHC of the parents has little or no effect on clutch size (Table S7). There is also weak support for non‐linear relationships since quadratic terms of functional divergence were retained in only one model (Tables S7 and S8). The MHC‐Iα functional divergence of each parent was included in two out of the three competing models with a ΔAICc ≤ 2, but no significant association was detected after model averaging (Figure 1a, Tables S7 and S8). For MHC‐IIβ DAB1, four out of five models with ΔAICc ≤ 2 included mothers' divergence, fathers' divergence, or both, but found no significant association after averaging the models (Figure 1a, Tables S7 and S8). For MHC‐IIβ DAB2, one out of the two models with ΔAICc ≤ 2 included the mothers' functional divergence (albeit not significantly in the average model, Figure 1a, Tables S7 and S8). The absolute values of the standardized effect sizes (β) of MHC divergence were of small magnitude, ranging from 0.006 to 0.031 and from 0.003 to 0.009 for the mother's and the father's functional divergence, respectively (see Figure 1a, Table S8, and Figure S4 for further details).

We found no association of clutch size with the presence/absence of any of the MHC supertypes of the parents or with the interaction of the presence/absence between the MHC supertypes of both parents (Table S9, Figure S5). All absolute β values were of small magnitude, ranging from 0.001 to 0.051 in the mother and from 0.001 to 0.058 in the father (see Table S9 and Figure S5 for further details).

### 3.3 Association of MHC divergence and supertypes with fledging success

When considering all types of nests (cross‐fostered and not cross‐fostered), our results do not support an association between fledging success and fathers' or mothers' functional divergence at any MHC loci. The interaction of the MHC‐IIβ DAB1 functional divergence of each parent was included in several models, although it was not significant in the average model. There was also weak support for non‐linear MHC associations (Tables S10 and S11). For MHC‐Iα, the four models with ΔAICc ≤ 2 included the fathers' and the mothers' functional divergence (Table S10), and although the fathers' divergence was included in all models, no significant effect was detected after model averaging (Figure 1b, Tables S10 and S11). Regarding MHC‐IIβ DAB1, there were six competing models, of which four included the interactive effects of the fathers' and the mothers' divergence, but no variable reached significance after model averaging (Figure 1b, Tables S10 and S11). From the MHC‐IIβ DAB2 models with ΔAICc ≤ 2, three out of four models included the mothers' or the fathers' functional divergence, but they were not significant in the averaged model (Tables S10 and S11). The absolute values of the standardized effect sizes (β) of MHC divergence were of small‐to‐moderate magnitudes, ranging from 0.024 to 0.135 and from 0.026 to 0.301 for the mother's and the father's functional divergence, respectively (see Figure 1b, Table S11, and Figure S6a,b for further details). However, the MHC‐IIβ DAB2 functional divergence showed the smallest magnitude of effect sizes on fledging success (0.024 for mothers and 0.026 for fathers; Figure 1b; Table S11; Figure S6c).

**FIGURE 2:**
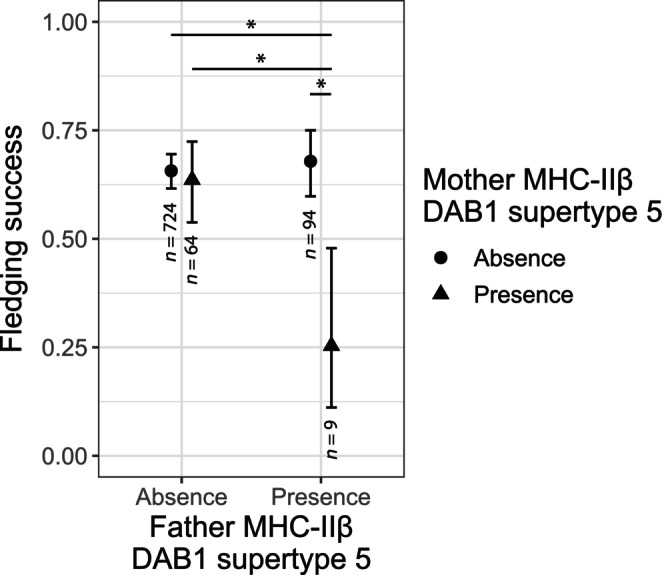
Association of the presence/absence of the MHC‐IIβ DAB1 supertype 5 with the fledging success of the chicks from different couple combinations. Each plot shows the estimated marginal means and 95% CI for each combination of parents regarding the presence of each supertype. The fledging success of couples in which both parents carry this supertype is significantly lower than in other couple combinations. Significance was calculated by performing a post‐hoc Tukey test: * significance at *p* < .05 (Table S13). The sample size for each couple combination regarding the presence/absence of this supertype is shown below the error bars.

We detected a significant interaction regarding the presence of MHC‐IIβ DAB1 supertype 5 on the fledging success (father x mother: β = −1.740 ± 0.548, *p* adjusted = 0.009; Table S12). *Post‐hoc* comparisons showed that when both parents carry the MHC‐IIβ DAB1 supertype 5 their chicks have a ca. 50% decrease in fledging success as compared to the other combinations of parental genotypes (Tukey tests: 5.154 < odds ratio < 6.234, 3.109 < z ratio < 3.495, 0.003 < *p* < .010; Figure 2; Table S13). Yet, this result needs to be taken cautiously because there are only 9 pairs (12 different individuals; mean number of fledglings per pair = 2) where both parents carried this specific supertype. No other supertype (paternal, maternal, or their interaction) showed evidence of association with fledging success (Table S12, Figure S7). The absolute values of the β of the presence/absence of the most common supertypes were of small‐to‐moderate magnitude and ranged from 0.007 to 0.452 and from 0.019 to 0.327 in the mother and the father, respectively (see Table S12 and Figure S7 for further details).

### 3.4 Genetic versus rearing effects of MHC divergence and supertypes on fledging success

**FIGURE 3:**
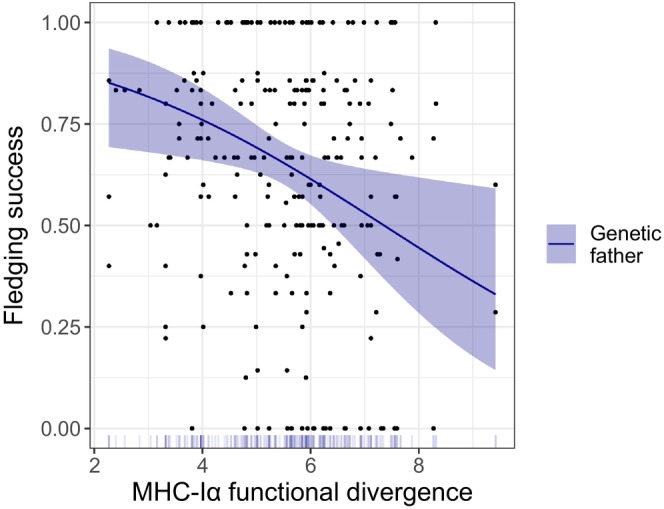
Fledging success as a function of the genetic fathers' MHC‐Iα functional divergence. The fitted regression lines are obtained from the estimated marginal means of the averaged GLMM model using the dataset subject to cross‐fostering (see methods section for modeling structure; and Tables S14 and S15 for more details). Dots represent the nests included in the analysis (*n* = 243). The significant negative slope of this relationship indicates that the more divergent the genetic father is at the MHC‐Iα genes, the fewer chicks survive until fledging.

Based only on cross‐fostered data, we detected a negative association between the MHC‐Iα functional divergence of the genetic father and fledging success (Figure 1c, Table S15, Figure S8). No other MHC terms (linear, quadratic, or in interaction), either of the genetic or social parents, showed evidence to significantly associate with the fledging success (Tables S14 and S15). In the four competing models of MHC‐Iα with ΔAICc ≤ 2, the genetic fathers' functional divergence was included in all models, whereas the social mothers' divergence was present in three models (Table S14). The average model showed that the functional divergence of genetic fathers was significantly associated with the proportion of chicks that fledged, with a moderate effect size (β = −0.461 ± 0.188; Table S15). Genetic fathers with less divergent alleles at MHC‐Iα produced chicks with higher fledging success than fathers with higher divergence (Figure 3). For MHC‐IIβ DAB1, there were eight competing models, and although the divergence of the social mother was present in seven out of eight models, no MHC‐IIβ DAB1 variable was significantly associated with fledging success (Figure 1c, Tables S14 and S15). Lastly, for the MHC‐IIβ DAB2, there were 11 models with ΔAICc ≤ 2, which denotes the uncertainty (Table S14) and included mainly both the genetic and social father functional divergences, but none was significant after model averaging (Tables S11 and S12). Concomitantly with the results with the whole dataset, the absolute values of the standardized effect sizes (β) were of small‐to‐moderate magnitudes, ranging from 0.067 to 0.500, from 0.024 to 0.461, from 0.158 to 0.320, and from 0.013 to 0.015, for the genetic mother's, genetic father's, social mother's, and social father's functional divergence, respectively (see Figure 1c, Table S15, and Figure S8 for further details). However, the effect sizes of MHC‐IIβ DAB2 functional divergences were of generally smaller magnitude, ranging from 0.013 (social father) to 0.094 (genetic father; see Figure 1c, Table S15, and Figure S8e,f for further details).

**FIGURE 4:**
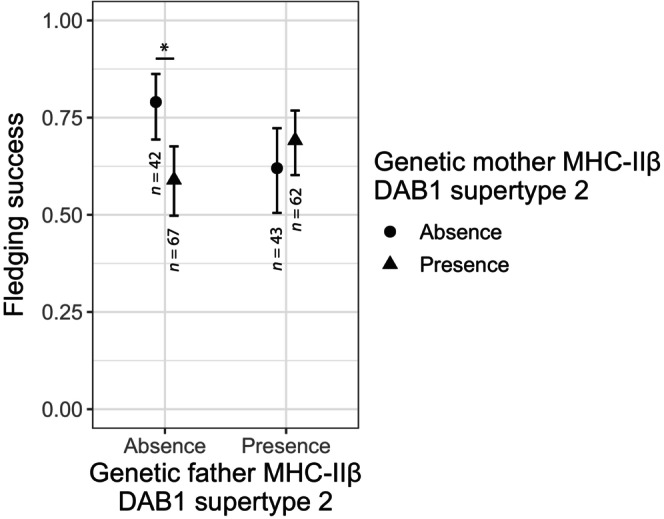
Association of the presence/absence of the MHC‐IIβ DAB1 supertype 2 with the fledging success of the chicks from different couple combinations. The plot shows the estimated marginal means and 95% CI for each combination of parents regarding the presence of each supertype. The fledging success of couples that do not carry this supertype is significantly higher than when only the genetic mother carries it. Significance was calculated by performing a post‐hoc Tukey test: * significance at *p* < .05 (Table S17). The sample size for each couple combination regarding the presence/absence of this supertype is shown below the error bars.

**FIGURE 5:**
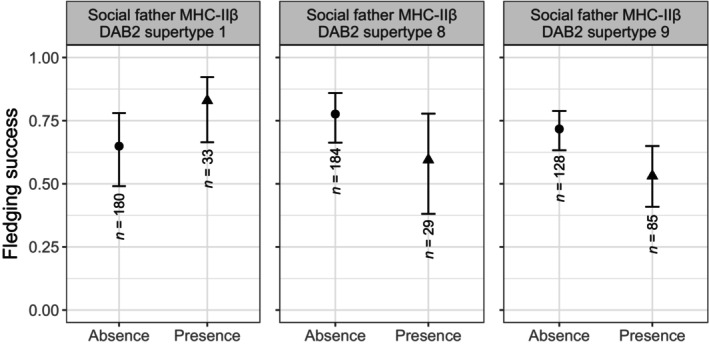
Association of the social father's MHC‐IIβ DAB2 supertypes 1, 8, and 9 with fledging success. The plot shows the estimated marginal means and 95% CI for the presence/absence of each supertype. The sample sizes, regarding the presence/absence of each supertype, are shown below the error bars.

We detected a significant interaction of the presence of MHC‐IIβ DAB1 supertype 2 in the genetic parents (father x mother: β = 1.280 ± 0.417, *p* adjusted = 0.013; Table S16). *Post‐hoc* comparisons between couples showed that only two combinations were significantly different: when both the genetic parents do not carry this supertype, their chicks' fledging success is ca. 25% higher than when only the female carries it (Tukey test: odds ratio = 2.618 ± 0.767, z ratio = 3.285, *p* = .006; Figure 4; Table S17). In addition, three MHC‐IIβ DAB2 supertypes of the social father were associated with the fledging success: carrying the supertype 1 increased the fledging success (β = 0.751 ± 0.348, *p* adjusted = 0.041; Figure 5; Table S16), while carrying supertypes 8 (β = −0.762 ± 0.347, *p* adjusted = 0.041; Figure 5; Table S16) or supertype 9 (β = −0.700 ± 0.257, *p* adjusted = 0.026; Figure 5; Table S16) decreased it. No other supertype showed evidence of association with fledging success, neither in the genetic nor in the social parents (Table S16; Figures S9 and S10). The absolute values of the β effect sizes of the supertypes on the fledging success achieved small‐to‐moderate magnitudes ranging from 0.029 to 1.069, from 0.001 to 0.636, from 0.004 to 0.676, and from 0.001 to 0.762 for the genetic mother's, genetic father's, social mother's, and social father's supertypes, respectively (see Table S16 and Figure S9 for further details).

## 4 DISCUSSION

In this study, we showed that parental MHC in barn owls seems to not be associated with the clutch size but to relate with fledging success via both MHC functional divergence and specific MHC supertypes. In line with our main hypothesis that the effect of MHC on reproductive success would depend on the combination of both parents' MHC, our study suggests that the presence of two MHC‐IIβ DAB1 supertypes has a conditional effect on the fledging success. We also detected a negative effect of MHC‐Iα divergence of the genetic father on fledging success, irrespective of the mother's MHC, and effects of three different MHC‐IIβ DAB2 supertypes of the social father. Finally, based on the results from a cross‐fostered dataset, we speculate that these effects on fledging success might reflect better parental care provided by fathers before hatching (from the genetic father) and until fledging (from the social father).

### 4.1 Parental MHC does not associate with clutch size

We did not find evidence for an association of clutch size with genetic variation at the MHC loci of fathers and mothers or by the combined genetic MHC diversity of the couple. The effect sizes of MHC variables were overall of small magnitude. This suggests that MHC diversity has none or little impact on reproductive investment in terms of the number of eggs produced. Since, in principle, MHC should influence individuals' health status and condition and thereby their capacity to invest in reproduction (Apanius et al., 1997), we expected an association between clutch size and at least the mothers' MHC diversity. Two different reasons could explain the lack of association between MHC and the clutch size in the barn owl. First, females may have limited contact with pathogens during egg rearing inside the nest, and thus energetic allocation on egg laying is barely affected. In line with this argument, female house sparrows (*Passer domesticus*) only altered the clutch size when immune challenged (Bonneaud et al., 2004). Second, the effect of MHC might be outweighed by the effect of other factors that are most likely the major drivers of clutch size, such as weather conditions and prey availability (Chausson et al., 2014; Durant et al., 2010). Overall, these findings are in line with previous studies in birds reporting no evidence for an association between MHC and clutch size (Sawada et al., 2020; Sepil, Lachish, & Sheldon, 2013b; Sun et al., 2019). As an exception, it was observed in the Eurasian coot (*Fulica atra*) that individuals with low and intermediate levels of MHC‐I and MHC‐II diversity, respectively, laid larger clutches (Pikus et al., 2022). However, whether these effects are only additive at the couple level remains to be addressed. In peafowls (*Pavo cristatus*), captive females laid larger eggs when mated with males with higher diversity at MHC‐II loci (Hale et al., 2009), suggesting that reproductive traits other than clutch size (investment in egg size) may depend on the combination of MHC diversity within a couple.

### 4.2 Specific combinations of the mother and father MHC associated with fledging success

In agreement with our expectations, we detected interactions regarding the presence of two MHC‐IIβ DAB1 supertypes. This suggests that certain combinations of MHC compositions on parents can be associated with the couples' reproductive success. Based on the full dataset, carrying MHC‐IIβ DAB1 supertype 5 on both parents decreased the fledging success by about 50%. Perhaps when both parents transmit this supertype to the offspring, it produces a disadvantageous effect by decreasing survival, as previously reported in the red junglefowl (*Gallus gallus*) (Worley et al., 2010). However, as (i) the sample size is very small for this combination, and (ii) the number of couples with both parents carrying the supertype 5 is following the expectation under random mating, this result and its interpretation must be taken with caution.

Based on the dataset including only full cross‐fostered clutches, we observed that the fledging success is higher when neither genetic parent carries MHC‐IIβ DAB1 supertype 2 but is significantly lower when only the mother carries it (Figure 4). The effect of this supertype can be indirect, through advantageous supertypes in the offspring, or directly on the genetic parents by increasing their reproductive fitness. Since we do not possess MHC data of the offspring, we cannot rule out that the chicks that receive this supertype could be more likely to die before fledging. Consequently, we can only speculate that if carrying this supertype is disadvantageous for the female, it could trigger a different reproductive strategy by the male, for instance, investing less in the brood when paired with a lower‐quality female based on MHC. Such behavioral adjustment was previously observed in mandrills (*Mandryllus sphinx*), where females with a specific MHC supertype were less mate‐guarded by males because that supertype was associated with lower immune function in this population (Setchell et al., 2016). There is evidence that sexually selected plumage ornaments are correlated with MHC gene expression in birds (Sly et al., 2022) and act as honest signals for individual quality in mate discrimination (Bollmer et al., 2012). In the barn owl, males showed lower feeding rates and decreased brood survival when females were less spotted (a plumage trait that signals immune quality; Roulin, 1999, Roulin et al., 2001). Ultimately, using phenotypic cues related to disadvantageous MHC supertypes could be a strategy to avoid the transmission of maladaptive alleles to offspring (Zelano & Edwards, 2002). To test this and to fully disentangle between parental and offspring effects, we would need to genotype the offspring and examine their survival prospects against their MHC composition.

### 4.3 Only the MHC of the fathers associated with fledging success

We extended our analysis to understand the relative contribution of MHC from the genetic parents versus the social parents. Using a cross‐fostered subset, we observed moderate‐size effects of MHC‐Iα functional divergence and MHC‐IIβ DAB1 supertype 2 in the genetic parents, and several supertypes of MHC‐IIβ DAB2 in the social parents. Although 6% of the chicks were cross‐fostered shortly after hatching, this was for a very short time after hatching; consequently, we believe that we can discard the effect of parental care provided by genetic parents after cross‐fostering. Non‐mutually exclusively, the fledging success of the chicks can be a result of direct genetic benefits, a product of better parental care from the genetic parents before cross‐fostering, or a result of better parental care provided by the social parents after cross‐fostering.

Regarding MHC‐Iα, the average model showed that the father's MHC‐Iα divergence, irrespective of the mother's MHC divergence, was negatively associated with fledging success. As the individual MHC‐Iα divergence cannot be directly inherited by the offspring (Lenz et al., 2013), we speculate that the genetic father's MHC‐Iα divergence influences the fledging success via better parental duties during incubation (i.e., before cross‐fostering). This is not surprising because, in the barn owl, the male plays a crucial role during the incubation period since he feeds the female (Roulin, 1999). We have previously observed that the MHC of neither parent affects the clutch size; however, it is possible that it affects other egg traits such as weight (Hale et al., 2009) or hatching probability (Hoover et al., 2018) that would increase the chances of survival post‐hatching. Moreover, against our theoretical expectations, the genetic father's MHC‐Iα divergence showed a considerable negative effect on the fledging success. Theoretical models of pathogen‐mediated selection and most of the empirical evidence support that higher MHC diversity induces higher individual performance. However, our findings are in line with the growing evidence supporting negative associations between MHC divergence and fitness‐related traits (e.g., Acevedo‐Whitehouse et al., 2018; Gagnon et al., 2020; Ilmonen et al., 2007; Lukasch et al., 2017; Pikus et al., 2022). Three alternative hypotheses could explain why lower MHC divergence can result in higher fledging success. First, individuals with higher MHC diversity may suffer less from immunopathology due to the negative T‐cell selection process (Migalska et al., 2019; Nowak et al., 1992). Although this would be expected in species harboring exceptionally high levels of diversity (e.g., passerines; Biedrzycka et al., 2017), simpler MHC structures have also been shown to have important implications in life‐history traits (e.g., Baratti et al., 2012; Bateson et al., 2016; Hoover et al., 2018; Wallny et al., 2006); consequently, we cannot rule out this hypothesis for the barn owl. Second, it could be a by‐product of the effect of specific genotypes or alleles/supertypes, as previously highlighted in Charbonnel et al. (2010) or Worley et al. (2010). However, we tested the association of fledging success with the presence of each MHC‐Iα supertype, and we did not detect any effect, suggesting that the negative association found is not mediated by any groups of functionally similar alleles. Third, selective pressures imposed by various pathogens can be limited in this barn owl population. Due to the energetic costs of mounting immune responses, it is possible that carrying less divergent alleles is already the optimal level of divergence given the lower pressure imposed by a less diverse community of parasites (Goüy De Bellocq et al., 2008; Pikus et al., 2022; Radwan et al., 2014). Considering our results, we speculate that selection is acting against individuals with higher levels of functional divergence at MHC‐Iα loci through a decrease in reproductive fitness, potentially reflecting a trade‐off between current reproductive effort and self‐maintenance (Bonneaud et al., 2004; Roved et al., 2018). Further work highlighting the pathogen community in the barn owl should be done to tackle the underlying mechanisms of our results.

After cross‐fostering, the chicks are reared at their social nest, so their survival until fledging is affected by their genetic makeup and/or by their social parents' provisioning. Our results showed that social fathers carrying supertype 1 positively associated with the fledging success of the chicks, whereas carrying supertypes 8 and 9 had a negative impact. Since this effect is not driven by the MHC of the chicks, it most likely reflects the quality of the social father, which is expected since the male has a crucial role for the brood completion. Indeed, specific MHC diversity has been shown to associate individual fitness in terms of resistance to pathogens (e.g., Migalska et al., 2022; Phillips et al., 2018), survival (e.g., Bateson et al., 2016; Brouwer et al., 2010), but also in reproduction (e.g., Eizaguirre et al., 2009; Hoover et al., 2018; Sepil, Lachish, & Sheldon, 2013b). As previously observed in great tits, specific supertypes conferred resistance to *Plasmodium* infections, which later translated into associations with reproductive success and adult survival (Sepil, Lachish, Hinks, & Sheldon, 2013a; Sepil, Lachish, & Sheldon, 2013b). While we do not possess information on the pathogen community affecting this population, it is possible that these supertypes also mediate a trade‐off between reproductive effort and infection.

Given the breeding behavior of this species, it is not surprising to detect larger effect sizes of MHC diversity on fledging success rather than on clutch size. Our results suggest that the MHC composition of the father, whether it is genetic or social, has an important association with the fledging success of the chicks, probably through parental care. This agrees with other studies suggesting that reproductive success is mainly associated with the MHC of the parents: by providing specific parental care, rather than the MHC diversity of the offspring (e.g., Bonneaud et al., 2004; Hale et al., 2009; Hoover et al., 2018; Lenz et al., 2013; Roved et al., 2018). Our argument that MHC improves adult condition rather than nestling condition is also supported by a recent study aiming at understanding the association between MHC composition and immunocompetence on nestling barn owls that did not find such evidence (Gaigher et al., 2019). As nestlings stay in the nest boxes until the fledging stage, their growth and survival are entirely dependent on the quality of the parental care (food provisioning) rather than directly due to their own MHC genetic makeup (Roulin, 1999).

Here, we analyzed MHC diversity at the genotype level of several genes. Alternatively, the combination of alleles of each MHC gene into haplotypes may represent a relevant MHC proxy to link with individual fitness (e.g., Huang et al., 2022) or disease resistance (e.g., Scherman et al., 2021). Previous studies on this population were able to infer haplotypes using an extensive family dataset and revealed the tight linkage of MHC genes (Gaigher et al., 2016; Gaigher et al., 2018). However, recombinant haplotypes were also detected, and together with the potential gene copy number variation and the high number of alleles, prevented the reconstruction of individual haplotypes in our current dataset, which relies on lineages of adults. Further work on genotyping the MHC of offspring will help to infer robust haplotypes but also disentangle parents from offspring MHC effects.

### 4.4 Inconsistencies between datasets

We detected an inconsistency between the results yielded from the analyses with the entire dataset and the ones based only on the cross‐fostered dataset. Specifically, the significant effects of both the genetic and social fathers were only revealed in the analysis of the cross‐fostered dataset but not in the entire dataset. Two reasons could explain such a discrepancy. First, two‐thirds of our entire dataset was subject to cross‐fostering manipulations, which could have introduced biases in the detection of such effects on fledging success. Concretely, being reared in a different nest could have impacted the offspring differently as if they were reared in the nest of origin since their survival could be associated with their own MHC genotype (e.g., genetic effect, Brouwer et al., 2010) and/or be attributed to the parents MHC genotype (e.g., parental care effect, Roved et al., 2018). Although in the analysis with the entire dataset (approximately 900 broods), the MHC of the genetic parents did not attain the same significance levels as with the cross‐fostered dataset (approximately 220 broods), both analyses showed generally consistent trends. This also suggests that the effects observed are not purely genetic; otherwise, we would have detected them with the entire dataset. Second, the significant results only found in the cross‐fostered dataset could be the result of Type I errors due to multiple testing and the substantial sample size reduction between the two datasets (from 900 to 220 broods). However, to minimize the likelihood of Type I errors, we: (i) included in our models several covariates known to influence the fledging success (i.e., age and laying date); and (ii) applied a stringent multiple‐testing correction during the analyses of multiple supertypes. Although we relied on a relatively large dataset, similar to what is found in the literature (e.g., Nelson‐Flower et al., 2023; Przesmycka et al., 2023; Roved et al., 2018; Zhang et al., 2023), and despite our attempts to reduce such errors, we cannot entirely ignore the risk of false positives and consequently require caution with the interpretation of some of our results.

### 4.5 Conclusion

Our study provides some support to an association between MHC diversity and fledging success but not with clutch size in a natural population of barn owls. On the one hand, we observe that the impact of fathers' MHC‐Iα functional divergence and the presence of MHC‐IIβ DAB2 supertypes on the fledging success is independent of the effect of his partner, but on the other hand, there is some indication that conditional effects might be more relevant in terms of specific supertypes on each parent at the MHC‐IIβ DAB1 locus. Although our results and their interpretations must be taken with caution, our findings highlight that the fitness consequences of MHC diversity may be underestimated or even undetected unless reproductive success is analyzed at several proxies. The same holds when considering different MHC classes, suggesting the effects of each MHC gene may act differently upon different life‐history traits of organisms. Previous studies in the context of MHC showed that the effect size must be interpreted considering the complexity of the immune responses and is expected to be of small magnitude (Gaigher et al., 2019; Hoover et al., 2018; Kamiya et al., 2014; Winternitz et al., 2017). The magnitude of our effect sizes agrees with recent studies in the field (e.g., Pikus et al., 2022; Pineaux et al., 2020; Roved et al., 2018; Sepil, Lachish, & Sheldon, 2013b) and general expectations in ecological studies (Møller & Jennions, 2002). In addition, future work could take advantage of this long‐term dataset to further test whether barn owls are employing MHC‐based mating choices to improve reproductive success. Indeed, previous studies showed non‐random pairing concerning immunity status in the barn owl (Roulin, 1999). Finally, new avenues of research should focus on comprehensive pathogen screening in both adults and offspring, combined with haplotypic history, to shed light on the precise mechanisms underlying our outcomes.

## AUTHOR CONTRIBUTIONS

**Diana Ferreira:** Conceptualization (equal); data curation (lead); formal analysis (lead); visualization (lead); writing – original draft (lead); writing – review and editing (lead). **Luis M. San‐José Garcia:** Supervision (lead); validation (equal); writing – original draft (equal); writing – review and editing (lead). **Alexandre Roulin:** Funding acquisition (equal); resources (lead); validation (equal); writing – original draft (supporting); writing – review and editing (supporting). **Arnaud Gaigher:** Conceptualization (equal); supervision (lead); validation (equal); writing – original draft (equal); writing – review and editing (lead). **Luca Fumagalli:** Funding acquisition (lead); project administration (lead); resources (lead); supervision (lead); validation (equal); writing – original draft (equal); writing – review and editing (equal).

## CONFLICT OF INTEREST STATEMENT

The authors declare no conflicting interests.

### BENEFIT‐SHARING SECTION

The results of this study are publicly available through the data repositories stated above.

## Supporting information


Data S1.

Data S2.

Data S3.

## Data Availability

Previously identified MHC‐Iα and MHC‐IIβ sequences are available on GenBank (accession numbers: KX189198‐KX189343 for MHC‐I; MG595289‐MG595313 for MHC‐II DAB1; MG595314‐MG595330 for MHC‐II DAB2). Accession numbers for the new sequences found in this study: OR047111‐OR047126 for MHC‐Iα, OR047105‐OR047110 for MHC‐IIβ DAB1, and OR047100‐OR047104 for MHC‐IIβ DAB2. Datasets and R scripts are available on the Dryad repository (https://doi.org/10.5061/dryad.8931zcrxn).
