# Supplementary material for: Limited associations between MHC diversity and reproductive success in a bird species with biparental care
Source: Ecol Evol. 2024 Feb 20;14(2):e10950. doi: 10.1002/ece3.10950 (PMC10879840; doi:10.1002/ece3.10950)

Figure S1. Clustering of MHC-Iα alleles by Discriminant Analysis of Principal Components (DAPC), based on the physicochemical properties of translated amino acids inferred to comprise the Peptide Binding Region (PBR). a) Number of the most appropriate number of clusters given by the lowest BIC value after which it increases, in this case, it is K = 9. b) Clustering visualization retaining the first and second axes of the Discriminant analysis (DA) which are the ones retaining most of the variation observed (DA eigenvalues, bottom left).

a)

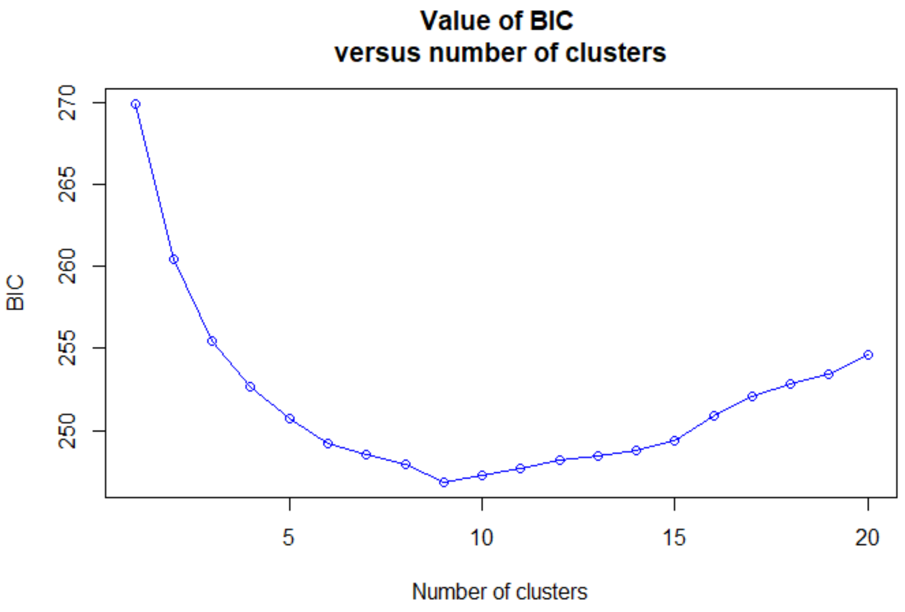

b)

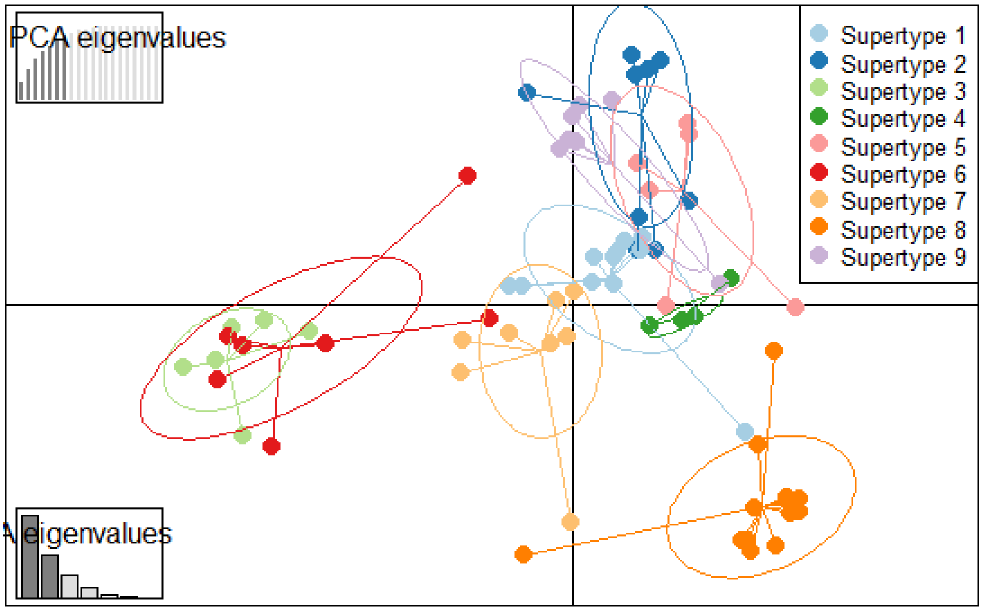

Figure S2. Clustering of MHC-IIβ DAB1 alleles by Discriminant Analysis of Principal Components (DAPC), based on the physicochemical properties of translated amino acids inferred to comprise the Peptide Binding Region (PBR). a) Number of the most appropriate number of clusters given by the highest average silhouette distance to other clusters, in this case, it is K = 14. b) Clustering visualization retaining the first and second axes of the Discriminant analysis (DA) which are the ones retaining most of the variation observed (DA eigenvalues, bottom right).

a)

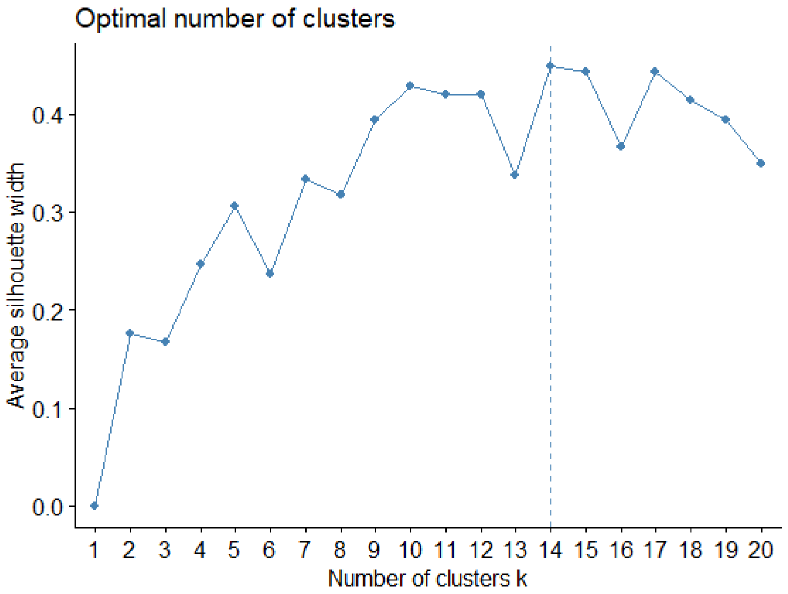

b)

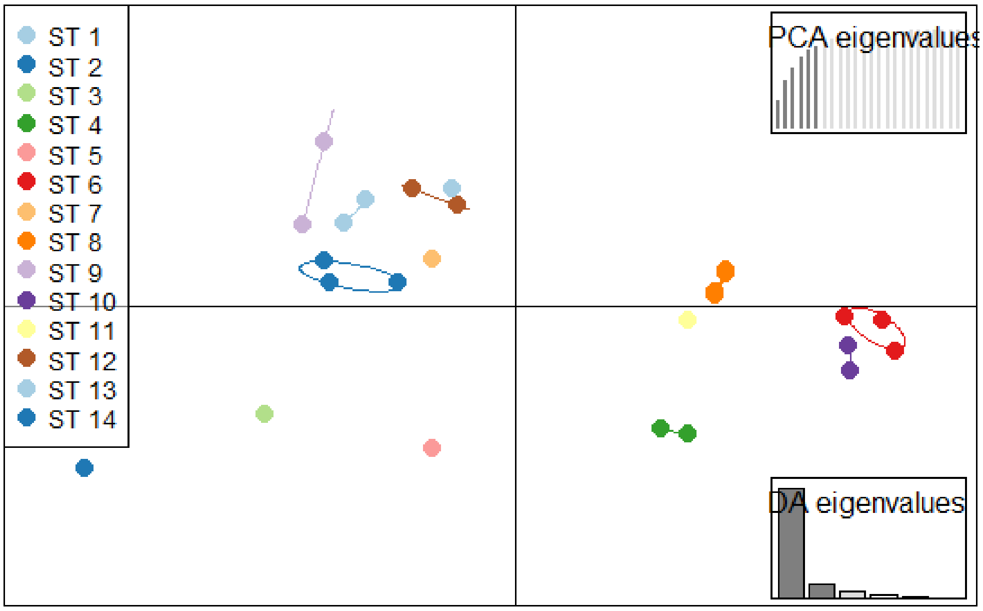

Figure S3. Clustering of MHC-IIβ DAB2 alleles by Discriminant Analysis of Principal Components (DAPC), based on the physicochemical properties of translated amino acids inferred to comprise the Peptide Binding Region (PBR). a) Number of the most appropriate number of clusters given by the highest average silhouette distance to other clusters, in this case, it is K = 10. b) Clustering visualization retaining the first and second axes of the Discriminant analysis (DA) which are the ones retaining most of the variation observed (DA eigenvalues, bottom left).

a)

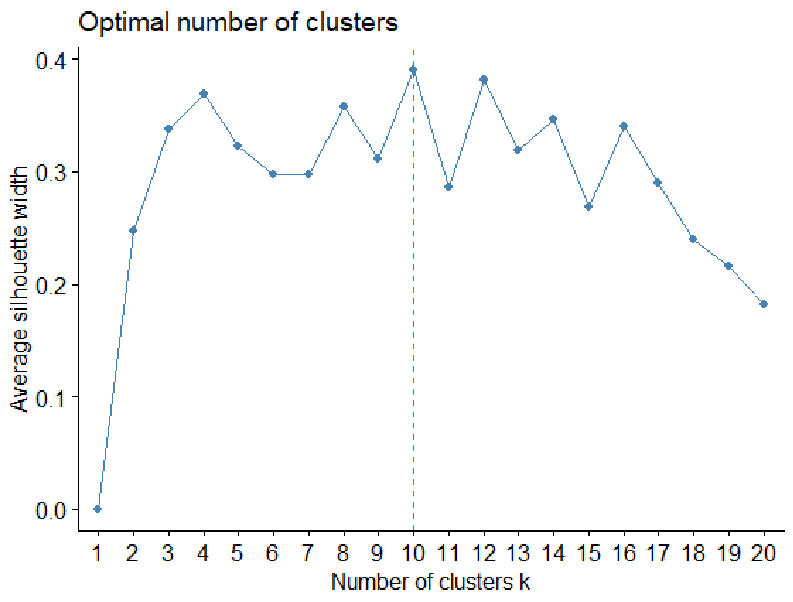

b)

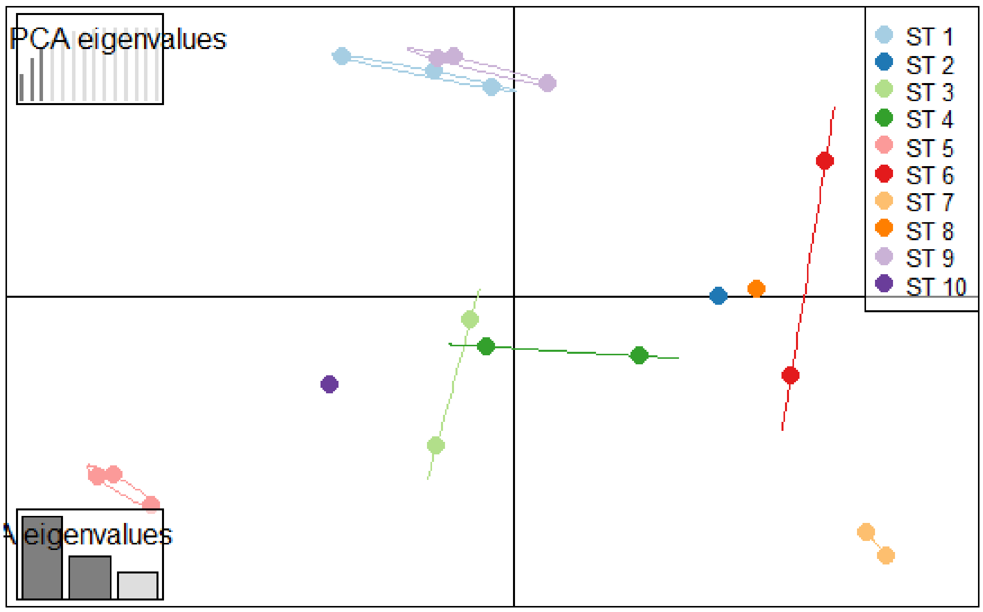

Figure S4. Clutch size as a function of the functional divergence between the alleles within A) the MHC-Iα; B) the MHC-IIβ DAB1; C) the MHC-IIβ DAB2. The fitted regression line is obtained from the estimated marginal means of the averaged GLMM model (see methods in the main text for modelling structure, and Table S7 and S8 for best models included and model averaging results). Each plot shows the predicted slope for the father's (dashed blue) and mother's (solid red) MHC functional divergence and respective confidence intervals while holding other co-variables at their mean.

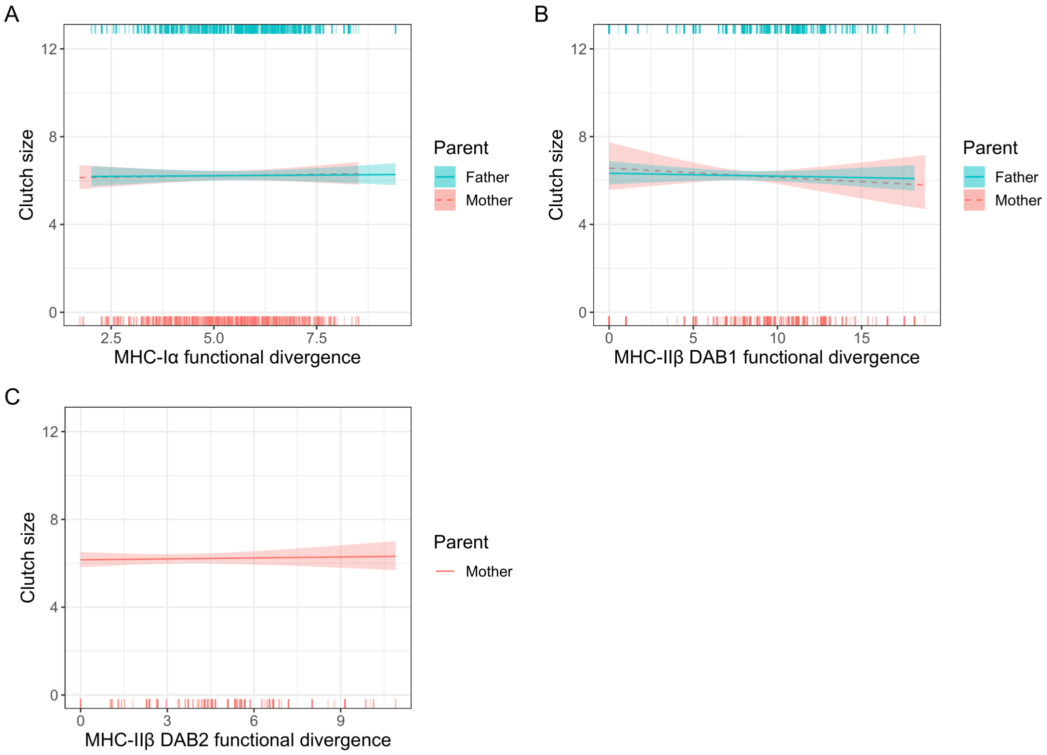

Figure S5. Estimated marginal means from GLMMs analysing the effect of the presence/absence of specific supertypes of A) MHC-Iα, B) MHC-IIβ DAB1; and C) MHC-IIβ DAB2 on the clutch size. Refer to the methods section in the main text for modelling structure; and Table S9 for simplified summary tables of models. Each plot shows the estimated marginal means and CI for each combination of parents regarding the presence of each supertype.

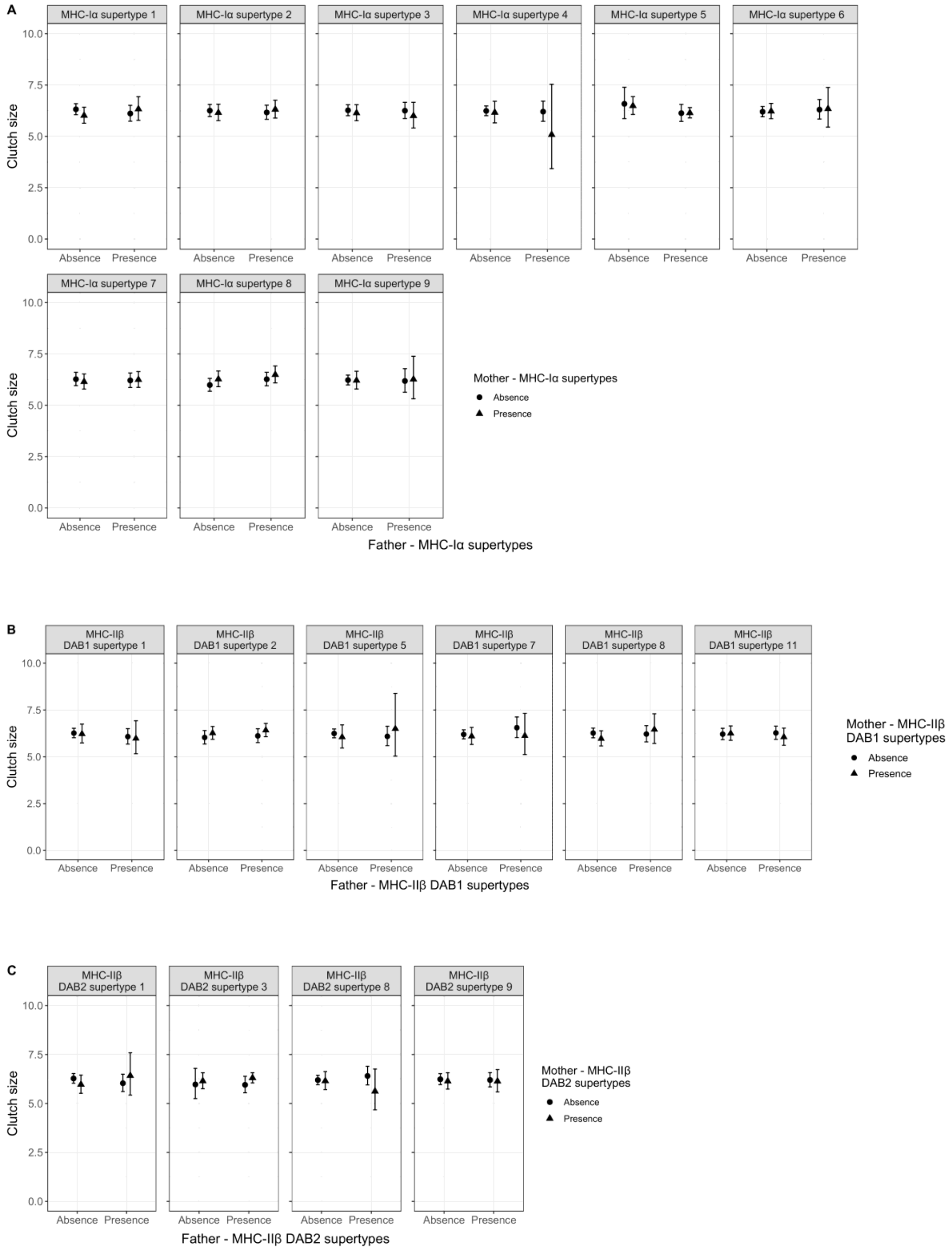

Figure S6. Fledging success as a function of the functional divergence between the alleles within A) the MHC-Iα; B) the MHC-IIβ DAB1; C) the MHC-IIβ DAB2. The fitted regression line is obtained from the estimated marginal means of the averaged GLMM model using the whole dataset (see methods in the main text for modelling structure, and Table S10 and S11 for best models included and model averaging results). Each plot shows the predicted slope for the father's (dashed blue) and mother's (solid red) MHC functional divergence and respective confidence intervals while holding other co-variables at their mean. The MHC functional divergence of either parent did not show evidence of explaining the observed fledging success and despite the apparent steep slopes, the wide confidence intervals convey the uncertainty of MHC divergence as an explaining factor of fledging success (see Discussion section).

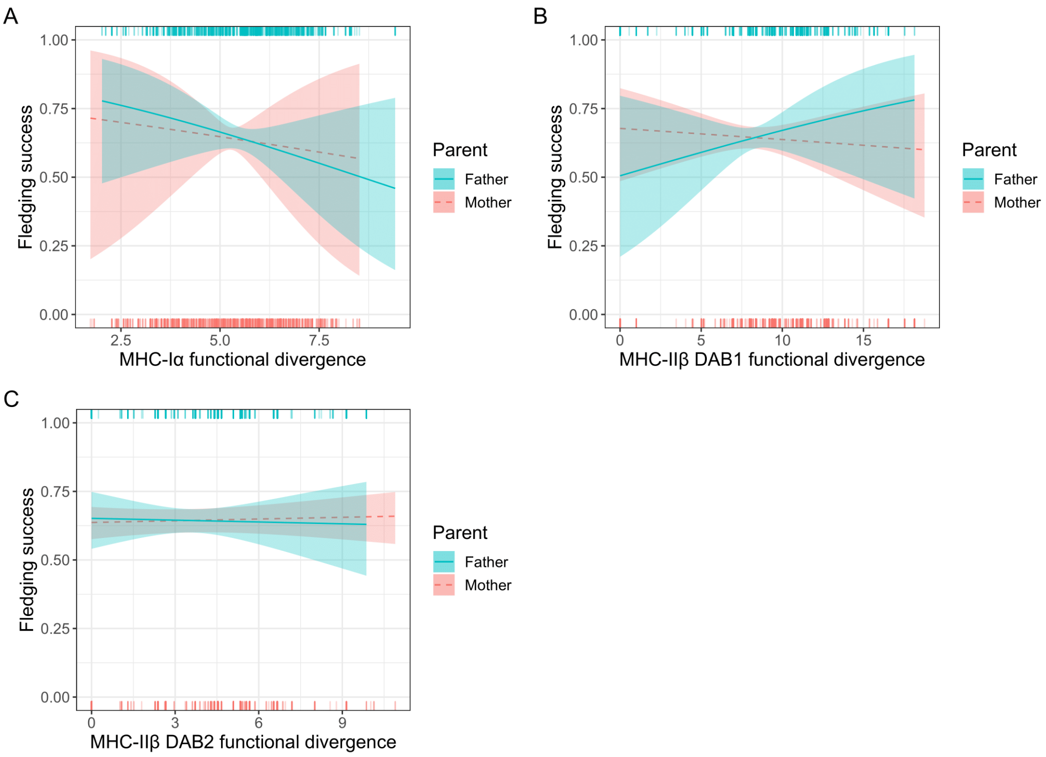

Figure S7. Estimated marginal means from GLMMs analysing the effect of the presence/absence of specific supertypes of A) MHC-Iα, B) MHC-IIβ DAB1; and C) MHC-IIβ DAB2 on fledging success. Refer to the methods section in the main text for modelling structure; and Table S12 for simplified summary tables of models. Each plot shows the estimated marginal means and CI for each combination of parents regarding the presence of each supertype. Supertype 5 of MHC-IIβ DAB1 (third panel on B) shows a significant decrease in the fledging success when both parents carry it (Table S13).

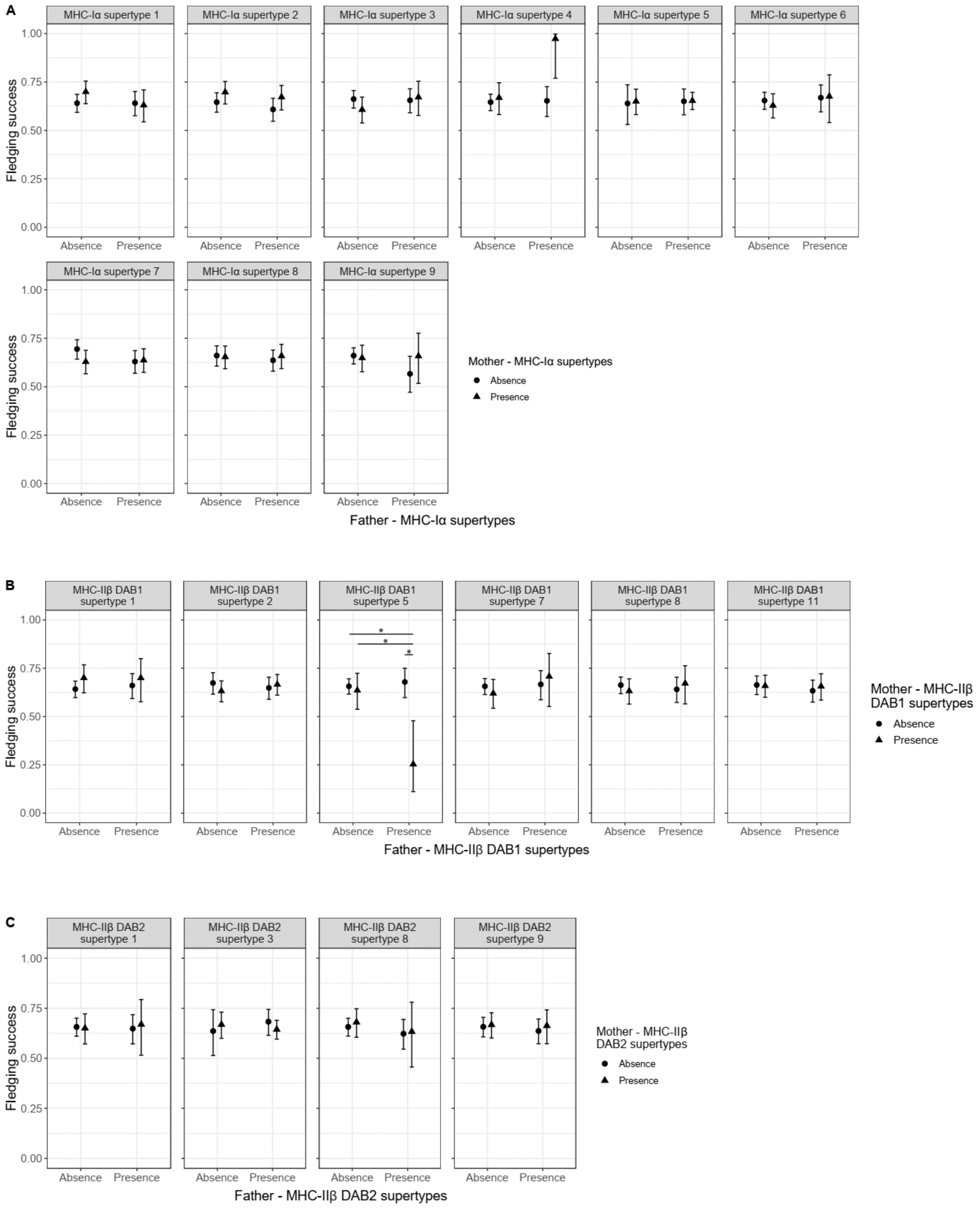

Figure S8. Fledging success as a function of the functional divergence between the alleles within A) and B) the MHC-Iα; C) and D) the MHC-IIβ DAB1; and E) and F) the MHC-IIβ DAB2. The fitted regression lines are obtained from the estimated marginal means of the averaged GLMM model using the dataset subject to cross-fostering (see methods in the main text for modelling structure, and Table S14 and S15 for best models included and model averaging results). The right-hand of the plots shows the predicted slopes for the MHC functional divergence and their CIs for the genetic parents and the left-hand for the social parents. The MHC-Iα functional divergence of the genetic father shows evidence of explaining the observed fledging success even though his chicks are raised in another nest (panel A; Table S15). The genetic mother's MHC-Iα functional divergence is not shown since it is not included in the top models (Table S15).

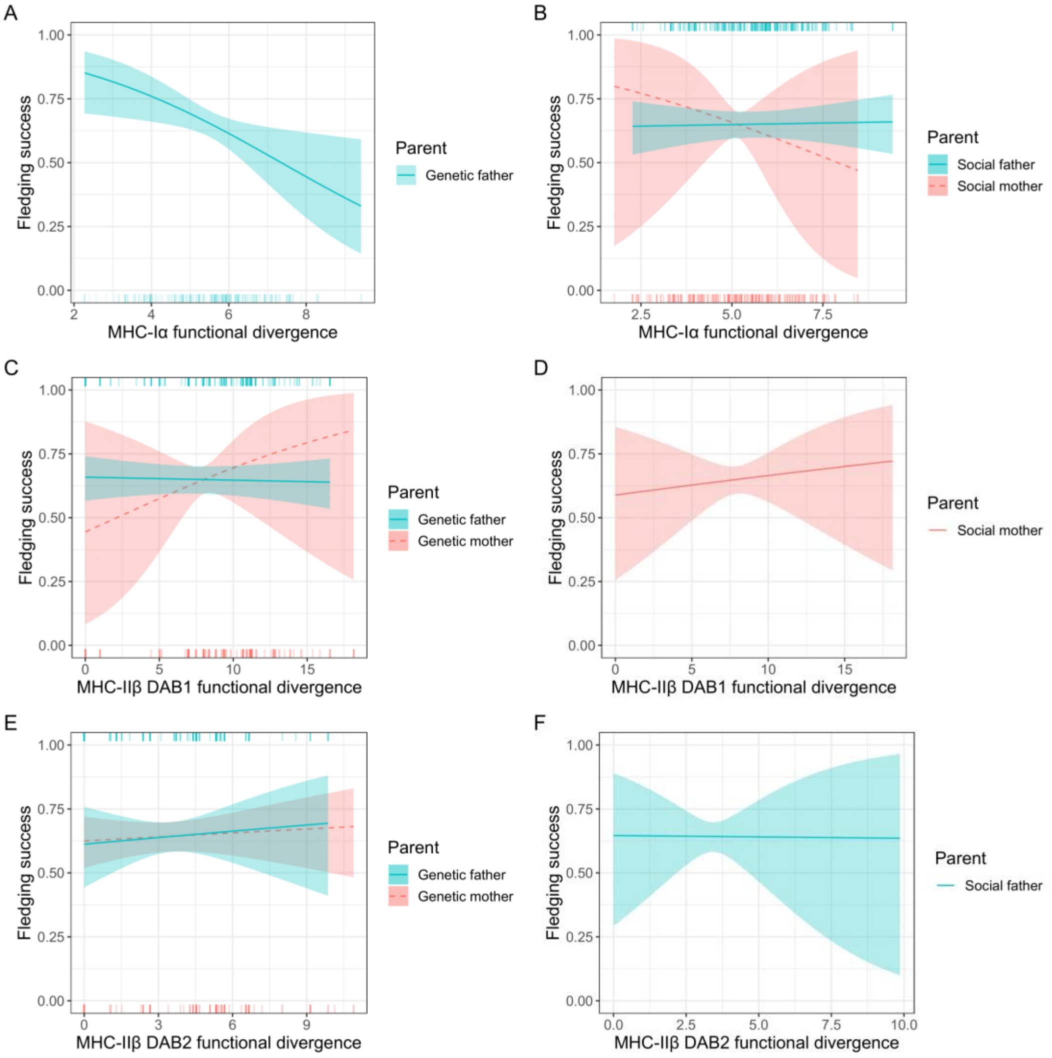

Figure S9. Estimated marginal means from GLMMs analysing the effect of the presence/absence of specific supertypes of A) MHC-Iα, B) MHC-IIβ DAB1; and C) MHC-IIβ DAB2 on the **genetic** parents on the fledging success. Refer to the methods section in the main text for modelling structure; and Table S16 for simplified summary tables of models. Each plot shows the estimated marginal means and CI for each combination of genetic parents regarding the presence of each supertype. Supertype 2 of MHC-IIβ DAB1 (second panel of B) shows a significant decrease in the fledging success when only the mother carries this supertype, as compared to when neither parent carries it (Table S17). The supertype 4 of MHC-Iα cannot be modelled with interaction due to low sample size, thus only the main effects are modelled and plotted.

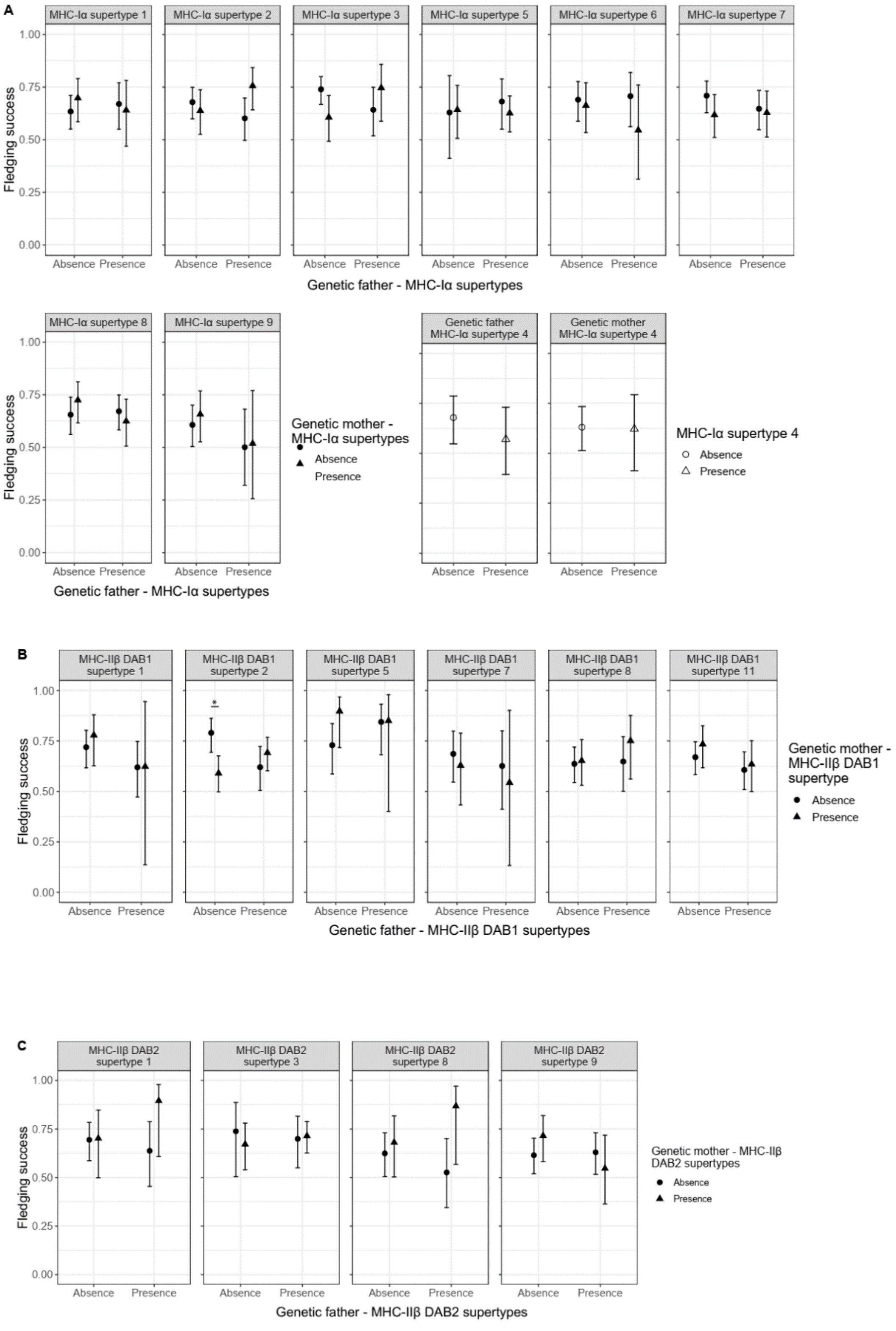

Figure S10. Estimated marginal means from GLMMs analysing the effect of the presence/absence of specific supertypes of A) MHC-Iα, B) MHC-IIβ DAB1; and C) MHC-IIβ DAB2 on the **social** parents on the fledging success. Refer to the methods section in the main text for modelling structure; and Table S16 for simplified summary tables of models. Each plot shows the estimated marginal means and CI for each combination of genetic parents regarding the presence of each supertype. Supertype 4 of MHC-Iα and supertype 1 of MHC-IIβ DAB1 cannot be modelled with interactions due to low sample size, thus only the main effects are modelled and plotted.

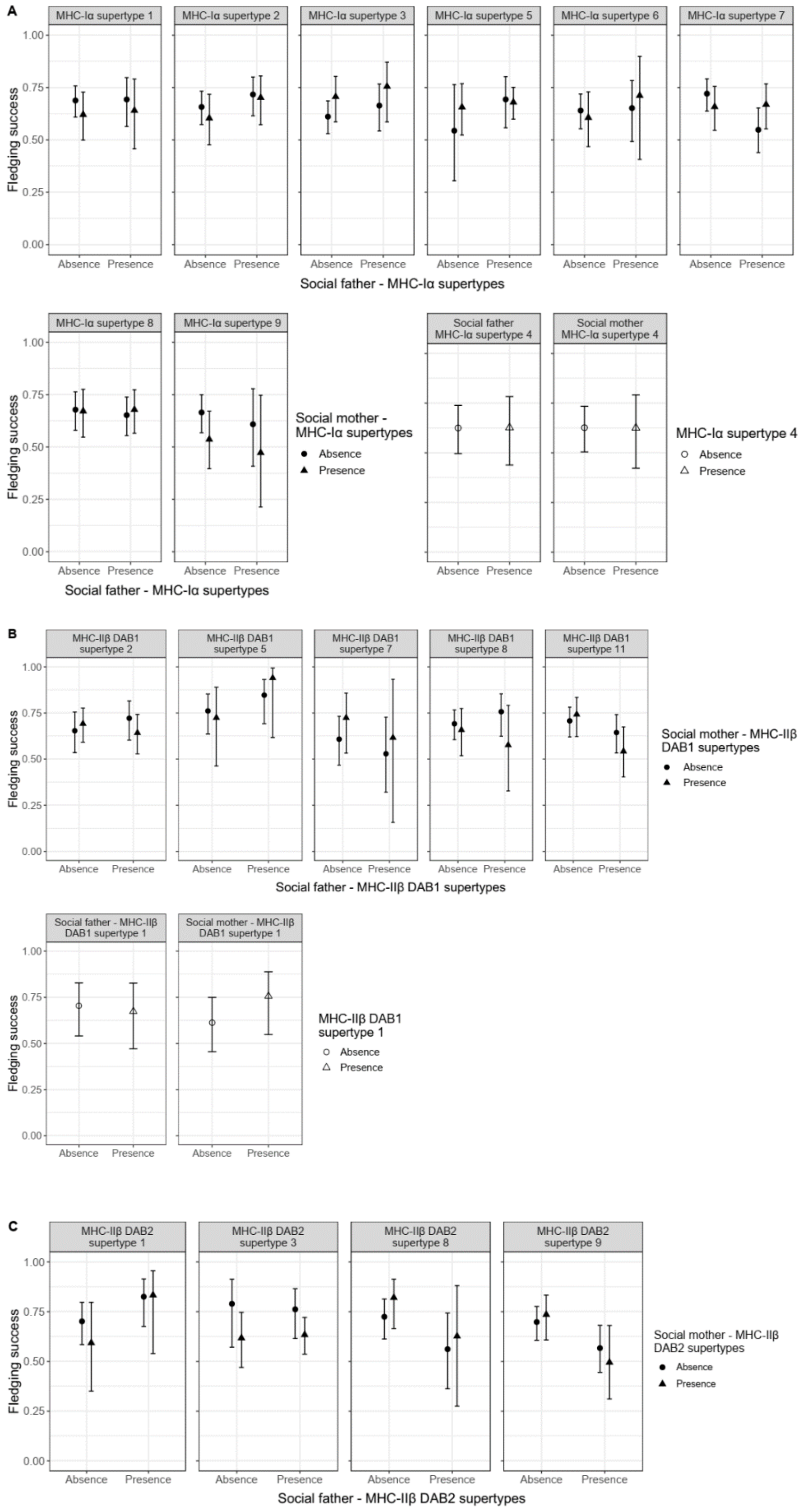

Supplement: Supplementary file 2 — Data S2. [file ECE3-14-e10950-s003.pdf]
