# Supplementary material for: Limited associations between MHC diversity and reproductive success in a bird species with biparental care
Source: Ecol Evol. 2024 Feb 20;14(2):e10950. doi: 10.1002/ece3.10950 (PMC10879840; doi:10.1002/ece3.10950)
Supplement: Supplementary file 3 — Data S3. [file ECE3-14-e10950-s001.pdf]

Table S1. Sample size of breeding attempts used per year for clutch size and fledging success analysis of each gene. Nb. obs: number of breeding attempts; ♀: number of different females; ♂: number of different males. Total values correspond to the total number of breeding attempts analysed and the total number of different females and males included.

	**Clutch size**									**Fledging success**								
	**MHC-Iα**			**MHC-IIβ DAB1**			**MHC-IIβ DAB2**			**MHC-Iα**			**MHC-IIβ DAB1**			**MHC-IIβ DAB2**		
Year	Nb obs	♀	♂	Nb obs	♀	♂	Nb obs	♀	♂	Nb obs	♀	♂	Nb obs	♀	♂	Nb obs	♀	♂
1994	1	1	1	1	1	1	1	1	1	1	1	1	1	1	1	1	1	1
1995	3	3	3	3	3	3	3	3	3	3	3	3	3	3	3	3	3	3
1996	5	5	5	5	5	5	5	5	5	5	5	5	5	5	5	5	5	5
1997	10	9	9	10	9	9	10	9	9	10	9	9	10	9	9	10	9	9
1998	41	35	36	41	35	36	41	35	36	41	35	36	41	35	36	41	35	36
1999	34	33	33	33	32	32	30	30	30	34	33	33	33	32	32	30	30	30
2000	52	51	48	52	51	48	49	48	46	52	51	48	52	51	48	49	48	46
2001	46	46	45	46	46	45	44	44	43	46	46	45	46	46	45	44	44	43
2002	72	69	66	72	69	65	71	68	65	72	69	66	72	69	65	71	68	65
2003	39	39	39	38	38	38	38	38	38	39	39	39	38	38	38	38	38	38
2004	40	27	35	40	27	35	41	28	36	40	27	35	40	27	35	41	28	36
2005	43	37	41	39	34	38	40	35	39	43	37	41	39	34	38	40	35	39
2006	22	21	21	17	16	16	20	19	19	22	21	21	17	16	16	20	19	19
2007	60	40	47	56	37	45	57	38	45	60	40	47	56	37	45	57	38	45
2008	63	59	57	60	56	54	61	57	55	63	59	57	60	56	54	61	57	55
2009	22	19	18	22	19	18	20	18	16	22	19	18	22	19	18	20	18	16
2010	43	33	35	41	31	33	41	32	34	43	33	35	41	31	33	41	32	34
2011	52	39	36	47	35	32	49	37	34	52	39	36	47	35	32	49	37	34
2012	90	75	71	85	69	66	85	69	67	90	74	71	85	69	66	85	69	67
2013	6	6	6	6	6	6	6	6	6	6	6	6	6	6	6	6	6	6
2014	17	11	14	16	10	13	15	10	12	17	11	14	16	10	13	15	10	12
2015	62	41	42	60	39	41	58	39	39	62	41	42	60	39	41	58	39	39
2016	47	38	33	46	38	33	47	38	33	47	38	33	46	38	33	47	38	33
2017	65	60	61	59	55	56	61	57	58	65	60	61	59	55	56	61	57	58
Total	935	516	426	895	491	411	893	493	413	935	516	426	895	491	411	893	493	413

Table S2. Sample size of different breeding attempts used per year for fledging success analysis of each gene on the cross-fostered dataset. Nb. obs: number of breeding attempts; ♀: number of different females; ♂: number of different males. Total values correspond to the total number of breeding attempts analysed and the total number of different females and males included.

Year	MHC-Iα					MHC-IIβ DAB1					MHC-IIβ DAB2				
	Nb Obs	Genetic Parents		Social parents		Nb Obs	Genetic Parents		Social parents		Nb Obs	Genetic Parents		Social parents	
		♀	♂	♀	♂		♀	♂	♀	♂		♀	♂	♀	♂
1999	22	22	22	22	22	20	20	20	20	20	18	18	18	18	18
2002	1	1	1	1	1	-	-	-	-	-	1	1	1	1	1
2003	34	34	34	34	34	32	32	32	32	32	32	32	32	32	32
2006	15	15	15	15	15	8	8	8	8	8	11	11	11	11	11
2008	1	1	1	1	1	1	1	1	1	1	1	1	1	1	1
2009	5	5	5	5	5	5	5	5	5	5	4	4	4	4	4
2010	16	16	16	15	15	15	15	15	14	15	16	16	16	15	15
2011	23	21	19	19	19	19	17	15	16	17	20	18	16	16	17
2012	62	52	53	52	53	54	44	46	44	46	54	44	45	44	45
2013	2	2	2	2	2	2	2	2	2	2	2	2	2	2	2
2014	6	5	6	5	6	6	5	6	5	6	6	5	6	5	6
2015	10	10	10	10	10	9	9	9	9	9	8	8	8	8	8
2016	38	33	32	31	31	36	31	30	30	29	34	29	29	27	28
2017	8	8	8	8	8	7	7	7	7	7	6	6	6	6	6
Total	243	190	175	186	182	214	168	154	166	160	213	165	153	163	158

Table S3. Frequency of MHC-Iα alleles in the barn owl population of western Switzerland and their GenBank accession numbers. New alleles found in this population are shown in bold.

Allele MHC-Iα	Freq.	GenBank	Allele MHC-Iα	Freq.	GenBank
Tyal-UA*01	0.1620	KX189198	Tyal-UA*56	0.0061	KX189253
Tyal-UA*02	0.0858	KX189199	Tyal-UA*57	0.0104	KX189254
Tyal-UA*03	0.0459	KX189200	Tyal-UA*58	0.0023	KX189255
Tyal-UA*04	0.0396	KX189201	Tyal-UA*59	0.0016	KX189256
Tyal-UA*05	0.0432	KX189202	Tyal-UA*60	0.0068	KX189257
Tyal-UA*06	0.0306	KX189203	Tyal-UA*62	0.0014	KX189259
Tyal-UA*07	0.0410	KX189204	Tyal-UA*65	0.0041	KX189262
Tyal-UA*08	0.0471	KX189205	Tyal-UA*66	0.0005	KX189263
Tyal-UA*09	0.0275	KX189206	Tyal-UA*67	0.0034	KX189264
Tyal-UA*10	0.0374	KX189207	Tyal-UA*69	0.0011	KX189266
Tyal-UA*11	0.0281	KX189208	Tyal-UA*70	0.0043	KX189267
Tyal-UA*12	0.0279	KX189209	Tyal-UA*71	0.0016	KX189268
Tyal-UA*13	0.0311	KX189210	Tyal-UA*72	0.0005	KX189269
Tyal-UA*14	0.0335	KX189211	Tyal-UA*78	0.0009	KX189275
Tyal-UA*15	0.0207	KX189212	Tyal-UA*79	0.0018	KX189276
Tyal-UA*16	0.0254	KX189213	Tyal-UA*83	0.0016	KX189280
Tyal-UA*17	0.0288	KX189214	Tyal-UA*84	0.0077	KX189281
Tyal-UA*18	0.0205	KX189215	Tyal-UA*85	0.0023	KX189282
Tyal-UA*19	0.0263	KX189216	Tyal-UA*93	0.0018	KX189289
Tyal-UA*20	0.0169	KX189217	Tyal-UA*96	0.0034	KX189292
Tyal-UA*21	0.0176	KX189218	Tyal-UA*103	0.0011	KX189299
Tyal-UA*22	0.0077	KX189219	Tyal-UA*105	0.0025	KX189301
Tyal-UA*23	0.0070	KX189220	Tyal-UA*106	0.0002	KX189302
Tyal-UA*24	0.0072	KX189221	Tyal-UA*116	0.0005	KX189310
Tyal-UA*25	0.0047	KX189222	Tyal-UA*117	0.0041	KX189311
Tyal-UA*26	0.0038	KX189223	Tyal-UA*123	0.0007	KX189316
Tyal-UA*27	0.0038	KX189224	Tyal-UA*126	0.0002	KX189319
Tyal-UA*28	0.0065	KX189225	Tyal-UA*133	0.0016	KX189325
Tyal-UA*29	0.0063	KX189226	Tyal-UA*135	0.0011	KX189327
Tyal-UA*30	0.0065	KX189227	Tyal-UA*144	0.0002	KX189332
Tyal-UA*31	0.0034	KX189228	Tyal-UA*146	0.0002	KX189333
Tyal-UA*32	0.0025	KX189229	Tyal-UA*154	0.0002	KX189341
Tyal-UA*34	0.0045	KX189231	Tyal-UA*155	0.0002	KX189342
Tyal-UA*35	0.0052	KX189232	Tyal-UA*158	0.0023	OR047111
Tyal-UA*36	0.0007	KX189233	Tyal-UA*165	0.0009	OR047112
Tyal-UA*37	0.0043	KX189234	Tyal-UA*174	0.0005	OR047113
Tyal-UA*38	0.0077	KX189235	**Tyal-UA*179**	0.0011	OR047114
Tyal-UA*39	0.0016	KX189236	**Tyal-UA*180**	0.0005	OR047115
Tyal-UA*41	0.0020	KX189238	**Tyal-UA*181**	0.0002	OR047116
Tyal-UA*43	0.0056	KX189240	**Tyal-UA*182**	0.0005	OR047117
Tyal-UA*44	0.0027	KX189241	**Tyal-UA*183**	0.0002	OR047118
Tyal-UA*46	0.0011	KX189243	**Tyal-UA*184**	0.0005	OR047119
Tyal-UA*47	0.0018	KX189244	**Tyal-UA*185**	0.0005	OR047120
Tyal-UA*48	0.0113	KX189245	**Tyal-UA*186**	0.0007	OR047121
Tyal-UA*50	0.0043	KX189247	**Tyal-UA*187**	0.0002	OR047122
Tyal-UA*51	0.0005	KX189248	**Tyal-UA*188**	0.0002	OR047123
Tyal-UA*52	0.0020	KX189249	**Tyal-UA*189**	0.0002	OR047124
Tyal-UA*53	0.0063	KX189250	**Tyal-UA*190**	0.0002	OR047125
Tyal-UA*54	0.0034	KX189251	**Tyal-UA*191**	0.0002	OR047126

Table S4. Frequency of MHC-IIβ DAB1 and MHC-IIβ DAB2 alleles in the barn owl population of western Switzerland and their GenBank accession numbers. New alleles found in this population are shown in bold.

Allele MHC-IIβ DAB1	Freq.	GenBank	Allele MHC-IIβ DAB2	Freq.	GenBank
Tyal-DAB1*01	0.2981	MG595289	Tyal-DAB2*01	0.4886	MG595314
Tyal-DAB1*02	0.0132	MG595290	Tyal-DAB2*02	0.0274	MG595315
Tyal-DAB1*03	0.0733	MG595291	Tyal-DAB2*03	0.0389	MG595316
Tyal-DAB1*04	0.0716	MG595292	Tyal-DAB2*04	0.0828	MG595317
Tyal-DAB1*05	0.3250	MG595293	Tyal-DAB2*05	0.0663	MG595318
Tyal-DAB1*06	0.0426	MG595294	Tyal-DAB2*06	0.0570	MG595319
Tyal-DAB1*07	0.0686	MG595295	Tyal-DAB2*07	0.4740	MG595320
Tyal-DAB1*08	0.0153	MG595296	Tyal-DAB2*08	0.0072	MG595321
Tyal-DAB1*09	0.0434	MG595297	Tyal-DAB2*09	0.0139	MG595322
Tyal-DAB1*10	0.1460	MG595298	Tyal-DAB2*10	0.0135	MG595323
Tyal-DAB1*11	0.0106	MG595299	Tyal-DAB2*11	0.0106	MG595324
Tyal-DAB1*12	0.0021	OR047105	Tyal-DAB2*12	0.0046	MG595325
Tyal-DAB1*13	0.0034	MG595300	Tyal-DAB2*16	0.0152	MG595326
Tyal-DAB1*14	0.0004	MG595301	Tyal-DAB2*17	0.0025	MG595327
Tyal-DAB1*16	0.0192	MG595302	Tyal-DAB2*18	0.0055	MG595328
Tyal-DAB1*17	0.0247	MG595303	Tyal-DAB2*19	0.0004	MG595329
Tyal-DAB1*18	0.0081	OR047106	Tyal-DAB2*20	0.0013	MG595330
Tyal-DAB1*19	0.0055	MG595304	Tyal-DAB2*24	0.0008	OR047100
Tyal-DAB1*20	0.0102	MG595305	Tyal-DAB2*35	0.0038	OR047101
Tyal-DAB1*21	0.0055	MG595306	**Tyal-DAB2*36**	0.0072	OR047102
Tyal-DAB1*22	0.0009	MG595307	**Tyal-DAB2*37**	0.0038	OR047103
Tyal-DAB1*23	0.0136	MG595308	**Tyal-DAB2*38**	0.0013	OR047104
Tyal-DAB1*24	0.0021	MG595309			
Tyal-DAB1*25	0.0094	MG595310			
Tyal-DAB1*26	0.0055	MG595311			
Tyal-DAB1*27	0.0013	MG595312			
Tyal-DAB1*28	0.0004	MG595313			
Tyal-DAB1*33	0.0021	OR047107			
Tyal-DAB1*36	0.0004	OR047108			
**Tyal-DAB1*39**	0.0009	OR047109			
**Tyal-DAB1*41**	0.0004	OR047110			

Table S5. Summary of the analysis of positive selection. See the supplementary material and methods section for more details.

		M1a	M2a	M7	M8
**MHC-Iα**	lnL	-2419.923	-2195.530	-1779.633	-1668.356
	LTR	448.793		222.553	
**MHC-IIβ DAB1**	lnL	-1377.218	-1318.234	-1299.876	-1245.921
	LTR	107.752		107.991	
**MHC-IIβ DAB2**	lnL	-1083.836	-1048.290	-956.476	-929.643
	LTR	70.923		53.666	

Table S6. Summary of supertype diversity and respective total frequency (number of individuals carrying supertype/total individuals). Number alleles: number of alleles clustered within each supertype; Frequency: frequency of the supertype in the barn owl population of western Switzerland. Each individual carriy 1-4 MHC-Iα supertypes, 1-2 MHC-IIβ DAB1 supertypes, and 1-2 MHC-IIβ DAB2 supertypes.

Gene	Supertype	Number alleles	Frequency
MHC-Iα	Supertype 1	12	0.304
	Supertype 2	6	0.370
	Supertype 3	9	0.255
	Supertype 4	5	0.097
	Supertype 5	16	0.780
	Supertype 6	13	0.222
	Supertype 7	14	0.453
	Supertype 8	15	0.414
	Supertype 9	8	0.162
MHC-IIβ DAB1	Supertype 1	3	0.158
	Supertype 2	2	0.532
	Supertype 3	1	0.001
	Supertype 4	1	0.030
	Supertype 5	4	0.095
	Supertype 6	4	0.074
	Supertype 7	2	0.138
	Supertype 8	2	0.220
	Supertype 9	1	0.026
	Supertype 10	1	0.001
	Supertype 11	3	0.396
	Supertype 12	2	0.031
	Supertype 13	3	0.090
	Supertype 14	2	0.022
MHC-IIβ DAB2	Supertype 1	3	0.155
	Supertype 2	2	0.061
	Supertype 3	3	0.788
	Supertype 4	2	0.024
	Supertype 5	1	0.025
	Supertype 6	2	0.025
	Supertype 7	2	0.084
	Supertype 8	2	0.151
	Supertype 9	3	0.311
	Supertype 10	2	0.006

Table S7. Models with AICc ≤ 2 explaining the relationship between clutch size and functional divergence of the mother and the father at MHC-Iα, MHC-IIβ DAB1 and MHC-IIβ DAB2 genes. The age of the individuals, and first and second-order levels of laying date were fixed in all models (including the null model) and were omitted for simplification (see section 2.6). df: degrees of freedom; AICc: Akaike information criterion with correction for small sample sizes; ΔAICc: difference in AICc to the top model with the lowest AICc; w: Akaike weight for each candidate model, as the probability of each model being the best model in the set. ER: a measure of how much better one model explains the data than the next model.

**MHC-Iα**					
**Model**	**df**	**AICc**	**ΔAICc**	**w**	**ER**
Null Model	8	3831.04	0.00	0.361	
Mother divergence	9	3832.37	1.33	0.186	1.94
Father divergence	9	3832.84	1.80	0.147	1.27

**MHC-IIβ DAB1**					
**Model**	**df**	**AICc**	**ΔAICc**	**w**	**ER**
Null Model	8	3675.12	0.00	0.196	
Mother divergence	9	3675.16	0.04	0.191	1.03
Mother divergence + Father divergence	10	3676.05	0.93	0.123	1.55
Father divergence	9	3676.14	1.02	0.117	1.05
Mother divergence + Mother divergence^2	10	3676.37	1.25	0.104	1.13

**MHC-IIβ DAB2**					
**Model**	**df**	**AICc**	**ΔAICc**	**w**	**ER**
Null Model	8	3662.06	0.00	0.388	
Mother divergence	9	3663.58	1.51	0.182	2.13

Table S8. Summary of GLMMs analyses that tested the influence of functional divergence on the clutch size of barn owls. The standardized estimates are unconditionally averaged from the models within the top two units of AICc model ranking (Table S7). Estimates are standardized in two SE and presented with 95% Confidence intervals (lower and upper CI). The response variable (clutch size) has errors under the Poisson distribution with a *logLink* function.

	Clutch size				
	**Variables**	**β estimate**	**SE**	**Lower CI**	**Upper CI**
**MHC-Iα** (n = 935)	Intercept	1.829	0.017	1.796	1.862
	Age mother	0.042	0.028	-0.013	0.097
	Age father	-0.045	0.029	-0.102	0.012
	Laying date	-0.153	0.176	-0.498	0.191
	Laying date^2	0.211	0.174	-0.130	0.552
	Mother divergence	0.006	0.017	-0.027	0.039
	Father divergence	0.003	0.013	-0.022	0.028
**MHC-IIβ DAB1** (n = 895)	Intercept	1.828	0.018	1.793	1.863
	Age mother	0.047	0.029	-0.010	0.104
	Age father	-0.053	0.029	-0.110	0.004
	Laying date	-0.174	0.179	-0.525	0.177
	Laying date^2	0.230	0.177	-0.117	0.577
	Mother divergence	-0.031	0.047	-0.123	0.061
	Mother divergence^2	0.010	0.036	-0.061	0.081
	Father divergence	-0.009	0.020	-0.048	0.030
**MHC-IIβ DAB2** (n = 893)	Intercept	1.825	0.018	1.790	1.860
	Age mother	0.045	0.029	-0.012	0.102
	Age father	-0.049	0.029	-0.106	0.008
	Laying date	-0.138	0.180	-0.491	0.215
	Laying date^2	0.190	0.178	-0.159	0.539
	Mother divergence	0.006	0.018	-0.029	0.041

Table S9. Summary of the models testing the influence of the presence/absence of specific supertypes on clutch size. Ages and laying dates were fixed in all models (see methods). Significance values (P value) were adjusted according to Benjamini-Hochberg procedure (Yoav & Yosef, 1995) to correct for multiple testing (P adj.). Fa: father; mo: mother; LD: laying date.

	**MHC-Iα ST1**			**MHC-Iα ST2**			**MHC-Iα ST3**			**MHC-Iα ST4**		
	**Estimate ± SE**	**P value**	**P adj**	**Estimate ± SE**	**P value**	**P adj**	**Estimate ± SE**	**P value**	**P adj**	**Estimate ± SE**	**P value**	**P adj**
**Intercept**	1.827 ± 0.018	0.000		1.827 ± 0.018	0.000		1.828 ± 0.018	0.000		1.827 ± 0.018	0.000	
**Father**	-0.010 ± 0.029	0.736	0980	-0.001 ± 0.027	0.980	0.980	-0.008 ± 0.030	0.791	0.980	-0.025 ± 0.043	0.555	0.980
**Mother**	-0.025 ± 0.030	0.394	0.980	0.001 ± 0.028	0.972	0.980	-0.026 ± 0.030	0.389	0.980	-0.035 ± 0.046	0.445	0.980
**LD**	-0.158 ± 0.198	0.427	0.453	-0.150 ± 0.200	0.453	0.453	-0.154 ± 0.199	0.439	0.453	-0.159 ± 0.199	0.425	0.453
**LD^2**	0.215 ± 0.193	0.265	0.283	0.209 ± 0.194	0.281	0.283	0.212 ± 0.194	0.274	0.283	0.216 ± 0.194	0.266	0.283
**Age fa**	-0.047 ± 0.029	0.100	0.265	-0.044 ± 0.029	0.127	0.148	-0.044 ± 0.029	0.121	0.148	-0.045 ± 0.029	0.116	0.148
**Age mo**	0.040 ± 0.029	0.158	0.265	0.040 ± 0.029	0.163	0.189	0.040 ± 0.029	0.168	0.189	0.041 ± 0.028	0.153	0.189
**Fa x mo**	0.084 ± 0.064	0.189	0.974	0.039 ± 0.056	0.487	0.974	-0.020 ± 0.069	0.770	0.974	-0.187 ± 0.209	0.373	0.974

	**MHC-Iα ST5**			**MHC-Iα ST6**			**MHC-Iα ST7**			**MHC-Iα ST8**		
	**Estimate ± SE**	**P value**	**P adj**	**Estimate ± SE**	**P value**	**P adj**	**Estimate ± SE**	**P value**	**P adj**	**Estimate ± SE**	**P value**	**P adj**
**Intercept**	1.827 ± 0.018	0.000		1.828 ± 0.018	0.000		1.828 ± 0.018	0.000		1.828 ± 0.018	0.000	
**Father**	-0.058 ± 0.032	0.067	0.542	0.017 ± 0.036	0.638	0.980	0.001 ± 0.027	0.962	0.980	0.041 ± 0.026	0.120	0.542
**Mother**	-0.001 ± 0.032	0.965	0.980	0.004 ± 0.031	0.893	0.980	-0.008 ± 0.027	0.761	0.980	0.041 ± 0.027	0.125	0.980
**LD**	-0.158 ± 0.199	0.427	0.453	-0.155 ± 0.199	0.437	0.453	-0.151 ± 0.199	0.448	0.453	-0.155 ± 0.199	0.436	0.453
**LD^2**	0.219 ± 0.193	0.258	0.283	0.213 ± 0.194	0.271	0.283	0.210 ± 0.194	0.279	0.283	0.213 ± 0.194	0.272	0.283
**Age fa**	-0.047 ± 0.029	0.099	0.148	-0.044 ± 0.029	0.132	0.148	-0.044 ± 0.029	0.126	0.148	-0.052 ± 0.029	0.074	0.148
**Age mo**	0.041 ± 0.029	0.150	0.189	0.041 ± 0.029	0.157	0.189	0.041 ± 0.029	0.152	0.189	0.042 ± 0.029	0.138	0.189
**Fa x mo**	0.017 ± 0.075	0.819	0.974	0.003 ± 0.090	0.974	0.974	0.026 ± 0.054	0.632	0.974	-0.012 ± 0.054	0.818	0.974

	**MHC-Iα ST9**			**MHC-IIβ DAB1 ST1**			**MHC-IIβ DAB1 ST2**			**MHC-IIβ DAB1 ST5**		
	**Estimate ± SE**	**P value**	**P adj**	**Estimate ± SE**	**P value**	**P adj**	**Estimate ± SE**	**P value**	**P adj**	**Estimate ± SE**	**P value**	**P adj**
**Intercept**	1.828 ± 0.018	0.000		1.827 ± 0.018	0.000		1.828 ± 0.018	0.000		1.828 ± 0.018	0.000	
**Father**	-0.004 ± 0.043	0.924	0.980	-0.032 ± 0.033	0.339	0.866	0.019 ± 0.027	0.475	0.866	-0.017 ± 0.042	0.681	0.866

**Mother**	-0.001 ± 0.034	0.980	0.980	-0.009 ± 0.038	0.817	0.817	0.043 ± 0.027	0.111	0.664	-0.020 ± 0.050	0.685	0.817
**LD**	-0.149 ± 0.199	0.453	0.453	-0.176 ± 0.203	0.388	0.434	-0.186 ± 0.203	0.359	0.434	-0.176 ± 0.202	0.383	0.434
**LD^2**	0.208 ± 0.194	0.283	0.283	0.232 ± 0.198	0.242	0.276	0.241 ± 0.197	0.222	0.276	0.231 ± 0.197	0.239	0.276
**Age fa**	-0.046 ± 0.029	0.114	0.148	-0.048 ± 0.030	0.104	0.104	-0.052 ± 0.029	0.078	0.104	-0.052 ± 0.029	0.076	0.104
**Age mo**	0.041 ± 0.028	0.146	0.189	0.047 ± 0.029	0.104	0.135	0.044 ± 0.029	0.135	0.135	0.046 ± 0.029	0.112	0.135
**Fa x mo**	0.016 ± 0.101	0.875	0.974	-0.009 ± 0.092	0.919	0.919	0.010 ± 0.054	0.854	0.919	0.097 ± 0.146	0.509	0.919

	**MHC-IIβ DAB1 ST7**			**MHC-IIβ DAB1 ST8**			**MHC-IIβ DAB1 ST11**			**MHC-IIβ DAB2 ST1**		
	**Estimate ± SE**	**P value**	**P adj**	**Estimate ± SE**	**P value**	**P adj**	**Estimate ± SE**	**P value**	**P adj**	**Estimate ± SE**	**P value**	**P adj**
**Intercept**	1.827 ± 0.018	0.000		1.827 ± 0.018	0.000		1.827 ± 0.018	0.000		1.825 ± 0.018	0.000	
**Father**	0.048 ± 0.040	0.232	0.866	0.011 ± 0.033	0.738	0.866	-0.005 ± 0.027	0.866	0.866	-0.022 ± 0.036	0.546	0.872
**Mother**	-0.022 ± 0.037	0.545	0.817	-0.031 ± 0.032	0.346	0. 817	-0.010 ± 0.028	0.719	0.817	-0.031 ± 0.038	0.410	0.613
**LD**	-0.158 ± 0.203	0.435	0.434	-0.180 ± 0.202	0.375	0.434	-0.165 ± 0.202	0.414	0.434	-0.142 ± 0.205	0.488	0.508
**LD^2**	0.215 ± 0.197	0.277	0.276	0.236 ± 0.197	0.231	0.276	0.222 ± 0.197	0.260	0.276	0.195 ± 0.200	0.330	0.350
**Age fa**	-0.050 ± 0.030	0.089	0.104	-0.053 ± 0.030	0.072	0.104	-0.051 ± 0.029	0.081	0.104	-0.051 ± 0.030	0.084	0.112
**Age mo**	0.045 ± 0.029	0.127	0.135	0.046 ± 0.029	0.114	0.135	0.045 ± 0.029	0.126	0.135	0.045 ± 0.029	0.123	0.147
**Fa x mo**	-0.052 ± 0.106	0.622	0.919	0.087 ± 0.079	0.266	0.919	-0.043 ± 0.058	0.459	0.919	0.112 ± 0.101	0.264	0.527

	**MHC-IIβ DAB2 ST3**			**MHC-IIβ DAB2 ST8**			**MHC-IIβ DAB2 ST9**		
	**Estimate ± SE**	**P value**	**P adj**	**Estimate ± SE**	**P value**	**P adj**	**Estimate ± SE**	**P value**	**P adj**
**Intercept**	1.825 ± 0.018	0.000		1.825 ± 0.018	0.000		1.825 ± 0.018	0.000	
**Father**	0.019 ± 0.032	0.549	0.872	0.013 ± 0.036	0.723	0.872	-0.005 ± 0.029	0.872	0.872
**Mother**	0.051 ± 0.034	0.132	0.527	-0.028 ± 0.037	0.460	0.613	-0.014 ± 0.031	0.644	0.853
**LD**	-0.136 ± 0.205	0.506	0.508	-0.152 ± 0.205	0.457	0.508	-0.136 ± 0.206	0.508	0.508
**LD^2**	0.186 ± 0.200	0.350	0.350	0.200 ± 0.199	0.315	0.350	0.188 ± 0.201	0.348	0.350
**Age fa**	-0.047 ± 0.030	0.112	0.112	-0.051 ± 0.030	0.088	0.112	-0.050 ± 0.030	0.092	0.112
**Age mo**	0.043 ± 0.029	0.147	0.147	0.048 ± 0.029	0.102	0.147	0.046 ± 0.029	0.121	0.147
**Fa x mo**	0.028 ± 0.082	0.727	0.932	-0.123 ± 0.108	0.255	0.527	0.006 ± 0.066	0.932	0.932

Table S10. Models with AICc ≤ 2 for the relationship between fledging success and functional divergence at MHC-Iα, MHC-IIβ DAB1 and MHC-IIβ DAB2. The age of the individuals, and first and second-order levels of laying date were fixed in all models (including the null model) and were omitted for simplification (see section 2.6). df: degrees of freedom; AICc: Akaike information criterion with correction for small sample sizes; ΔAICc: difference in AICc to the top model with the lowest AICc; w: Akaike weight for each candidate model, as the probability of each model being the best model in the set. ER: a measure of how much better one model explains the data than the next model.

**MHC-Iα**						
**Model**		**df**	**AICc**	**ΔAICc**	**w**	**ER**
1	Father divergence	9	3626.99	0.00	0.268	
2	Mother divergence + Father divergence	10	3627.88	0.89	0.172	1.56
3	Mother divergence + Mother divergence^2 + Father divergence	11	3628.18	1.18	0.148	1.16
4	Father divergence + Father divergence^2	10	3628.97	1.98	0.100	1.48

**MHC-IIβ DAB1**						
**Model**		**df**	**AICc**	**ΔAICc**	**w**	**ER**
1	Mother divergence + Father divergence + Father divergence^2 + Mother divergence x Father divergence	12	3479.82	0.00	0.187	
2	Father divergence + Father divergence^2	10	3480.03	0.21	0.168	1.11
3	Mother divergence + Mother divergence^2 + Father divergence + Father divergence^2 + Mother divergence x Father divergence	13	3480.34	0.52	0.144	1.16
4	Null Model	8	3481.03	1.21	0.102	1.41
5	Mother divergence + Father divergence + Mother divergence x Father divergence	11	3481.33	1.51	0.088	1.16
6	Mother divergence + Mother divergence^2 + Father divergence + Mother divergence x Father divergence	12	3481.80	1.98	0.069	1.28

**MHC-IIβ DAB2**						
**Model**		**df**	**AICc**	**ΔAICc**	**w**	**ER**
1	Null Model	8	3477.07	0.00	0.257	
2	Mother divergence	9	3478.17	1.10	0.148	1.74
3	Father divergence	9	3478.59	1.52	0.120	1.23
4	Father divergence + Father divergence^2	10	3479.02	1.95	0.097	1.24

Table S11. Summary of GLMMs analyses that tested the influence of functional divergence on the fledging success of barn owls. The standardized estimates are unconditionally averaged from the models within the top two units of AICc model ranking (Table S10). Estimates are standardized in two SE and presented with 95% Confidence intervals. The response variable has binomial distributed errors with a *logit* function. The terms highlighted are statistically significant.

	Fledging success				
	**Variables**	**β estimate**	**SE**	**Lower CI**	**Upper CI**
**MHC-Iα** (n = 935)	Intercept	0.580	0.090	0.404	0.756
	Age mother	0.276	0.111	0.058	0.494
	Age father	0.090	0.108	-0.122	0.302
	Laying date	-0.289	0.508	-1.285	0.707
	Laying date^2	-0.130	0.502	-1.114	0.854
	Mother divergence	-0.135	0.466	-1.048	0.778
	Mother divergence ^{2}	0.187	0.483	-0.760	1.134
	Father divergence	-0.266	0.268	-0.791	0.259
	Father divergence^2	-0.023	0.250	-0.513	0.467
**MHC-IIβ DAB1** (n = 895)	Intercept	0.596	0.088	0.424	0.768
	Age mother	0.239	0.112	0.019	0.459
	Age father	0.072	0.109	-0.142	0.286
	Laying date	-0.219	0.512	-1.223	0.785
	Laying date^2	-0.218	0.506	-1.210	0.774
	Mother divergence	-0.083	0.225	-0.524	0.358
	Mother divergence^2	0.102	0.230	-0.349	0.553
	Father divergence	0.301	0.357	-0.399	1.001
	Father divergence^2	-0.385	0.374	-1.118	0.348
	Mother divergence x Father divergence	0.236	0.226	-0.207	0.679
**MHC-IIβ DAB2** (n = 893)	Intercept	0.592	0.095	0.406	0.778
	Age mother	0.251	0.115	0.026	0.476
	Age father	0.087	0.113	-0.134	0.308
	Laying date	-0.512	0.522	-1.535	0.511
	Laying date^2	0.112	0.516	-0.899	1.123
	Mother divergence	0.024	0.069	-0.111	0.159
	Father divergence	-0.026	0.162	-0.344	0.292
	Father divergence^2	0.057	0.177	-0.290	0.404

Table S12. Summary of the models testing the influence of the presence/absence of specific supertypes on fledging success. Ages and laying dates were fixed in all models (see methods). Significance values (P value) were adjusted according to Benjamini-Hochberg procedure (Yoav & Yosef, 1995) to correct for multiple testing (P adj.). Fa: father; mo: mother; LD: laying date.

	**MHC-Iα ST1**			**MHC-Iα ST2**			**MHC-Iα ST3**			**MHC-Iα ST4**		
	**Estimate ± SE**	**P value**	**P adj**	**Estimate ± SE**	**P value**	**P adj**	**Estimate ± SE**	**P value**	**P adj**	**Estimate ± SE**	**P value**	**P adj**
**Intercept**	0.629 ± 0.091	0.000		0.622 ± 0.092	0.000		0.625 ± 0.091	0.000		0.652 ± 0.092	0.000	
**Father**	-0.087 ± 0.119	0.465	0.722	-0.145 ± 0.110	0.186	0.418	0.052 ± 0.124	0.677	0.762	0.327 ± 0.202	0.105	0.418
**Mother**	0.178 ± 0.121	0.143	0.387	0.253 ± 0.112	0.023	0.192	-0.155 ± 0.125	0.215	0.387	0.452 ± 0.223	0.043	0.192
**LD**	-0.297 ± 0.519	0.567	0.567	-0.336 ± 0.521	0.520	0.567	-0.349 ± 0.521	0.503	0.567	-0.307 ± 0.520	0.555	0.567
**LD^2**	-0.150 ± 0.514	0.770	0.851	-0.111 ± 0.516	0.830	0.851	-0.098 ± 0.516	0.849	0.851	-0.133 ± 0.515	0.796	0.851
**Age fa**	0.095 ± 0.110	0.386	0.475	0.106 ± 0.111	0.341	0.475	0.094 ± 0.111	0.396	0.475	0.084 ± 0.111	0.452	0.475
**Age mo**	**0.284 ± 0.112**	**0.011**	**0.014**	**0.286 ± 0.112**	**0.011**	**0.014**	**0.279 ± 0.113**	**0.014**	**0.014**	**0.279 ± 0.112**	**0.013**	**0.014**
**Fa x mo**	-0.310 ± 0.227	0.173	0.366	0.042 ± 0.201	0.835	0.897	0.308 ± 0.242	0.203	0.366	2.860 ± 1.235	0.021	0.185

	**MHC-Iα ST5**			**MHC-Iα ST6**			**MHC-Iα ST7**			**MHC-Iα ST8**		
	**Estimate ± SE**	**P value**	**P adj**	**Estimate ± SE**	**P value**	**P adj**	**Estimate ± SE**	**P value**	**P adj**	**Estimate ± SE**	**P value**	**P adj**
**Intercept**	0.627 ± 0.091	0.000		0.627 ± 0.092	0.000		0.627 ± 0.092	0.000		0.627 ± 0.091	0.000	
**Father**	0.021 ± 0.132	0.874	0.874	0.101 ± 0.144	0.482	0.722	-0.151 ± 0.108	0.162	0.418	-0.055 ± 0.108	0.607	0.762
**Mother**	0.020 ± 0.131	0.877	0.974	-0.088 ± 0.126	0.487	0.730	-0.144 ± 0.110	0.189	0.387	0.032 ± 0.111	0.773	0.974
**LD**	-0.336 ± 0.521	0.519	0.567	-0.335 ± 0.520	0.520	0.567	-0.349 ± 0.521	0.503	0.567	-0.337 ± 0.521	0.518	0.567
**LD^2**	-0.111 ± 0.516	0.830	0.851	-0.112 ± 0.515	0.829	0.851	-0.096 ± 0.515	0.852	0.851	-0.107 ± 0.515	0.835	0.851
**Age fa**	0.097 ± 0.111	0.384	0.475	0.106 ± 0.111	0.342	0.475	0.116 ± 0.110	0.293	0.475	0.103 ± 0.112	0.356	0.475
**Age mo**	**0.280 ± 0.113**	**0.013**	**0.014**	**0.286 ± 0.113**	**0.011**	**0.014**	**0.290 ± 0.112**	**0.009**	**0.014**	**0.281 ± 0.112**	**0.013**	**0.014**
**Fa x mo**	-0.035 ± 0.269	0.897	0.897	0.142 ± 0.321	0.658	0.846	0.325 ± 0.188	0.083	0.366	0.128 ± 0.190	0.499	0.748

	**MHC-Iα ST9**			**MHC-IIβ DAB1 ST1**			**MHC-IIβ DAB1 ST2**			**MHC-IIβ DAB1 ST5**		
	**Estimate ± SE**	**P value**	**P adj**	**Estimate ± SE**	**P value**	**P adj**	**Estimate ± SE**	**P value**	**P adj**	**Estimate ± SE**	**P value**	**P adj**
**Intercept**	0.621 ± 0.090	0.000		0.639 ± 0.088	0.000		0.640 ± 0.088	0.000		0.636 ± 0.086	0.000	
**Father**	-0.318 ± 0.171	0.063	0.418	0.070 ± 0.139	0.614	0.804	0.030 ± 0.107	0.782	0.804	-0.042 ± 0.170	0.804	0.804
**Mother**	-0.005 ± 0.143	0.974	0.974	0.246 ± 0.154	0.109	0.415	-0.053 ± 0.108	0.624	0.749	-0.291 ± 0.196	0.138	0.415
**LD**	-0.335 ± 0.519	0.519	0.567	-0.250 ± 0.524	0.634	0.634	-0.256 ± 0.524	0.625	0.634	-0.261 ± 0.521	0.617	0.634

**LD²**	-0.112 ± 0.514	0.827	0.851	-0.215 ± 0.519	0.678	0.735	-0.210 ± 0.520	0.685	0.735	-0.202 ± 0.517	0.696	0.735
**Age fa**	0.079 ± 0.111	0.475	0.475	0.067 ± 0.112	0.548	0.548	0.085 ± 0.111	0.440	0.548	0.081 ± 0.110	0.461	0.548
**Age mo**	**0.277 ± 0.112**	**0.014**	**0.014**	**0.230 ± 0.112**	**0.040**	**0.041**	**0.239 ± 0.113**	**0.034**	**0.041**	**0.231 ± 0.112**	**0.038**	**0.041**
**Fa x mo**	0.440 ± 0.339	0.194	0.366	-0.084 ± 0.322	0.795	0.795	0.263 ± 0.185	0.155	0.464	**-1.740 ± 0.548**	**0.002**	**0.009**

	MHC-IIβ DAB1 ST7			MHC-IIβ DAB1 ST8			MHC-IIβ DAB1 ST11			MHC-IIβ DAB2 ST1		
	**Estimate ± SE**	**P value**	**P adj**	**Estimate ± SE**	**P value**	**P adj**	**Estimate ± SE**	**P value**	**P adj**	**Estimate ± SE**	**P value**	**P adj**
**Intercept**	0.635 ± 0.089	0.000		0.637 ± 0.089	0.000		0.636 ± 0.088	0.000		0.642 ± 0.095	0.000	
**Father**	0.101 ± 0.159	0.525	0.804	-0.036 ± 0.131	0.786	0.804	-0.089 ± 0.111	0.420	0.804	-0.019 ± 0.151	0.900	0.900
**Mother**	-0.115 ± 0.150	0.443	0.749	-0.077 ± 0.128	0.546	0.749	0.028 ± 0.112	0.801	0.801	-0.007 ± 0.153	0.963	0.963
**LD**	-0.287 ± 0.526	0.585	0.634	-0.284 ± 0.525	0.588	0.634	-0.277 ± 0.525	0.598	0.634	-0.535 ± 0.533	0.315	0.320
**LD²**	-0.177 ± 0.521	0.735	0.735	-0.184 ± 0.520	0.724	0.735	-0.190 ± 0.520	0.715	0.735	0.109 ± 0.527	0.836	0.841
**Age fa**	0.090 ± 0.112	0.421	0.548	0.081 ± 0.111	0.465	0.548	0.081 ± 0.111	0.464	0.548	0.091 ± 0.115	0.427	0.455
**Age mo**	**0.230 ± 0.113**	**0.041**	**0.041**	**0.230 ± 0.113**	**0.041**	**0.041**	**0.232 ± 0.112**	**0.039**	**0.041**	**0.260 ± 0.116**	**0.025**	**0.026**
**Fa x mo**	0.350 ± 0.377	0.353	0.529	0.271 ± 0.268	0.312	0.529	0.120 ± 0.201	0.550	0.660	0.121 ± 0.360	0.737	0.879

	MHC-IIβ DAB2 ST3			MHC-IIβ DAB2 ST8			MHC-IIβ DAB2 ST9		
	**Estimate ± SE**	**P value**	**P adj**	**Estimate ± SE**	**P value**	**P adj**	**Estimate ± SE**	**P value**	**P adj**
**Intercept**	0.640 ± 0.095	0.000		0.641 ± 0.095	0.000		0.639 ± 0.095	0.000	
**Father**	-0.041 ± 0.135	0.760	0.900	-0.159 ± 0.150	0.291	0.900	-0.072 ± 0.122	0.552	0.900
**Mother**	-0.102 ± 0.135	0.449	0.775	0.096 ± 0.154	0.535	0.775	0.068 ± 0.123	0.581	0.775
**LD**	-0.548 ± 0.533	0.304	0.320	-0.529 ± 0.532	0.320	0.320	-0.555 ± 0.533	0.298	0.320
**LD²**	0.125 ± 0.527	0.813	0.841	0.105 ± 0.526	0.841	0.841	0.130 ± 0.527	0.806	0.841
**Age fa**	0.086 ± 0.115	0.455	0.455	0.089 ± 0.115	0.441	0.455	0.100 ± 0.115	0.387	0.455
**Age mo**	**0.259 ± 0.116**	**0.026**	**0.026**	**0.260 ± 0.116**	**0.025**	**0.026**	**0.260 ± 0.116**	**0.025**	**0.026**
**Fa x mo**	-0.317 ± 0.292	0.277	0.879	-0.061 ± 0.397	0.879	0.879	0.069 ± 0.234	0.767	0.879

Table S13. Post-hoc Tukey pairwise comparisons of the fledging success between all couples regarding the presence or absence of MHC-IIβ DAB1 supertype 5 in each parent. "0": absence, "1": presence; "♂": father, "♀": mother. Tukey adjustment for multiple comparisons was applied and tests were performed on the *log* odds ratio scale. Results are averaged over the other covariates.

Contrasts					Odds ratio	SE	*z* ratio	P value
♂	♀	–	♂	♀				
0	0	–	1	0	0.905	0.160	-0.565	0.943
0	0	–	0	1	1.095	0.225	0.440	0.972
**0**	**0**	**–**	**1**	**1**	**5.643**	**2.865**	**3.408**	**0.004**
1	0	–	0	1	1.120	0.315	0.730	0.885
**1**	**0**	**–**	**1**	**1**	**6.234**	**3.264**	**3.495**	**0.003**
**0**	**1**	**–**	**1**	**1**	**5.154**	**2.719**	**3.109**	**0.010**

Table S14. Models with AICc<2 for the relationship between fledging success and functional divergence of genetic and social parents at MHC-Iα, MHC-IIβ DAB1, and MHC-IIβ DAB2. The age of the individuals, and first and second-order levels of laying date were fixed in all models (including the null model) and were omitted for simplification (see section 2.6). df: degrees of freedom; AICc: Akaike information criterion with correction for small sample sizes; ΔAICc: difference in AICc to the top model with the lowest AICc; w: Akaike weight for each candidate model, as the probability of each model being the best model. ER: a measure of how much better one model explains the data than the next model. GFa: genetic father; GMo: genetic mother; SFa: social father; SMo: social mother.

**MHC-Iα**						
**Model**		**df**	**AICc**	**ΔAICc**	**w**	**ER**
1	GFa divergence + SMo divergence	14	928.78	0.00	0.103	
2	GFa divergence	13	929.19	0.41	0.084	1.23
3	GFa divergence + SMo divergence + SFa divergence	15	930.76	1.98	0.038	2.21
4	GFa divergence + SMo divergence + SMo divergence^2	15	930.76	1.98	0.038	1.00

**MHC-IIβ DAB1**						
**Model**		**df**	**AICc**	**ΔAICc**	**w**	**ER**
1	SMo divergence	13	825.73	0.00	0.058	
2	GMo divergence + GMo divergence^2 + SMo divergence	15	825.99	0.26	0.051	1.14
3	GMo divergence + GMo divergence^2	14	826.60	0.87	0.038	1.34
4	SMo divergence + SMo divergence^2	14	826.63	0.90	0.037	1.03
5	Null model divergence	12	826.99	1.26	0.031	1.19
6	GMo divergence + GMo divergence^2 + SMo divergence + SMo divergence^2	16	827.24	1.51	0.027	1.15
7	GFa divergence + SMo divergence	14	827.49	1.76	0.024	1.13
8	GMo divergence + GMo divergence^2 + GFa divergence + SMo divergence	16	827.67	1.94	0.022	1.09

**MHC-IIβ DAB2**						
**Model**		**df**	**AICc**	**ΔAICc**	**w**	**ER**
1	SFa divergence	13	824.05	0.00	0.058	
2	SFa divergence + SFa divergence^2	14	824.17	0.12	0.055	1.05
3	GFa divergence + SFa divergence	14	824.66	0.61	0.042	1.31
4	GFa divergence + SFa divergence + SFa divergence^2	15	824.98	0.94	0.036	1.17
5	GMo divergence + SFa divergence + SFa divergence^2	15	825.10	1.05	0.034	1.06
6	GMo divergence + SFa divergence	14	825.12	1.07	0.034	1.00
7	Null model	12	825.22	1.18	0.032	1.06
8	GFa divergence	13	825.33	1.29	0.031	1.03
9	GMo divergence + GFa divergence + SFa divergence	15	825.79	1.75	0.024	1.29
10	GMo divergence + GFa divergence + SFa divergence + SFa divergence^2	16	825.98	1.93	0.022	1.09
11	GFa divergence + GFa divergence^2 + SFa divergence	15	826.03	1.98	0.022	1.00

Table S15. Summary of GLMMs analyses that tested the influence of functional divergence on fledging success of barn owls. The standardized (β) estimates are unconditionally averaged from the models within the top two units of AICc model ranking (Table S14). Estimates are standardized in two SD and presented with 95% Confidence intervals (CI). The response variable has binomial distributed errors with a *logit* function. The terms highlighted are statistically significant.

	Fledging success – Genetic vs rearing effects				
	**Variables**	**β Estimate**	**SE**	**Lower CI**	**Upper CI**
**MHC-Iα** (n = 243)	Intercept	0.621	0.115	0.396	0.846
	Age genetic mother	0.106	0.202	-0.290	0.502
	Age genetic father	-0.091	0.201	-0.485	0.303
	Age social mother	-0.182	0.213	-0.599	0.235
	Age social father	0.573	0.224	0.134	1.012
	Laying date	1.182	1.126	-1.025	3.389
	Laying date^2	-1.401	1.118	-3.592	0.790
	Genetic father divergence	-0.461	0.188	-0.829	-0.093
	Social mother divergence	-0.320	0.629	-1.553	0.913
	Social mother divergence^2	0.097	0.573	-1.026	1.220
	Social father divergence	0.015	0.086	-0.154	0.184
**MHC-IIβ DAB1** (n = 214)	Intercept	0.617	0.121	0.380	0.854
	Age genetic mother	0.052	0.206	-0.352	0.456
	Age genetic father	0.043	0.204	-0.357	0.443
	Age social mother	-0.082	0.233	-0.539	0.375
	Age social father	0.475	0.241	0.003	0.947
	Laying date	0.928	1.176	-1.377	3.233
	Laying date^2	-1.161	1.165	-3.444	1.122
	Genetic mother divergence	0.500	0.655	-0.784	1.784
	Genetic mother divergence^2	-0.548	0.697	-1.914	0.818
	Genetic father divergence	-0.024	0.094	-0.208	0.160
	Social mother divergence	0.158	0.436	-0.697	1.013
	Social mother divergence^2	0.148	0.414	-0.663	0.959
**MHC-IIβ DAB2** (n = 213)	Intercept	0.586	0.129	0.333	0.839
	Age genetic mother	0.125	0.224	-0.314	0.564
	Age genetic father	0.001	0.214	-0.418	0.420
	Age social mother	-0.294	0.242	-0.768	0.180
	Age social father	0.519	0.251	0.027	1.011
	Laying date	0.638	1.233	-1.779	3.055
	Laying date^2	-0.751	1.219	-3.140	1.638
	Genetic mother divergence	0.067	0.157	-0.241	0.375
	Genetic father divergence	0.094	0.236	-0.369	0.557
	Genetic father divergence^2	0.029	0.176	-0.316	0.374
	Social father divergence	-0.013	0.562	-1.115	1.089
	Social father divergence^2	-0.328	0.577	-1.459	0.803

Table S16. Summary of the results from models testing the relationship between fledging success and the presence/absence of specific supertypes about the **genetic** parents and/or the **social** parents. Age, laying date and laying date^2 were fixed in all models (see methods). Significance values were adjusted according to Benjamini-Hochberg procedure (Yoav & Yosef, 1995) to correct for multiple testing. The terms highlighted are statistically significant. "-" indicates a lack of observations of certain levels of the interaction, thus interaction effect is not modelled. LD: laying date.

	MHC-Iα ST1			MHC-Iα ST2			MHC-Iα ST3			MHC-Iα ST4		
	**Estimate ± SE**	**P value**	**P adj**	**Estimate ± SE**	**P value**	**P adj**	**Estimate ± SE**	**P value**	**P adj**	**Estimate ± SE**	**P value**	**P adj**
**Intercept**	0.686 ± 0.122	0.000		0.659 ± 0.122	0.000		0.672 ± 0.119	0.000		0.690 ± 0.122	0.000	
**Genetic father**	0.035 ± 0.232	0.879	0.988	-0.035 ± 0.204	0.863	0. 988	-0.139 ± 0.222	0.531	0.956	-0.464 ± 0.285	0.104	0.625
**Genetic mother**	0.170 ± 0.232	0.462	0.614	0.156 ± 0.214	0.467	0. 614	-0.338 ± 0.219	0.122	0. 614	-0.037 ± 0.386	0.923	0.923
**Social father**	0.040 ± 0.254	0.876	0.983	0.328 ± 0.227	0.148	0.664	0.233 ± 0.244	0.339	0.851	0.007 ± 0.321	0.983	0.983
**Social mother**	-0.285 ± 0.245	0.245	0.734	-0.172 ± 0.241	0.474	0.953	0.431 ± 0.254	0.090	0.405	-0.005 ± 0.369	0.990	0.990
**LD**	1.130 ± 1.168	0.333	0.422	1.173 ± 1.165	0.314	0.422	0.911 ± 1.135	0.422	0.422	1.019 ± 1.156	0.378	0.422
**LD^2**	-1.347 ± 1.158	0.244	0.310	-1.360 ± 1.149	0.236	0.310	-1.090 ± 1.131	0.335	0.335	-1.250 ± 1.147	0.276	0.310
**Age gen. father**	-0.018 ± 0.209	0.931	0.948	-0.105 ± 0.203	0.604	0.948	-0.078 ± 0.200	0.698	0.948	-0.032 ± 0.210	0.878	0.948
**Age gen. mother**	0.124 ± 0.210	0.557	0.685	0.124 ± 0.210	0.557	0.685	0.083 ± 0.205	0.685	0.685	0.170 ± 0.210	0.419	0.685
**Age soc. father**	**0.537 ± 0.241**	**0.026**	**0.033**	**0.503 ± 0.241**	**0.037**	**0.037**	**0.585 ± 0.235**	**0.013**	**0.026**	**0.572 ± 0.241**	**0.018**	**0.026**
**Age soc. mother**	-0.204 ± 0.223	0.360	0.458	-0.213 ± 0.222	0.338	0.458	-0.176 ± 0.218	0.420	0.458	-0.205 ± 0.223	0.359	0.458
**Gen. father x mother**	-0.418 ± 0.499	0.402	0.619	0.904 ± 0.435	0.038	0.169	1.104 ± 0.486	0.023	0.169	-	-	-
**Soc. father x mother**	0.069 ± 0.542	0.898	1.000	0.165 ± 0.462	0.721	1.000	0.018 ± 0.515	0.972	1.000	-	-	-

	MHC-Iα ST5			MHC-Iα ST6			MHC-Iα ST7			MHC-Iα ST8		
	**Estimate ± SE**	**P value**	**P adj**	**Estimate ± SE**	**P value**	**P adj**	**Estimate ± SE**	**P value**	**P adj**	**Estimate ± SE**	**P value**	**P adj**
**Intercept**	0.689 ± 0.122	0.000		0.690 ± 0.123	0.000		0.673 ± 0.121	0.000		0.693 ± 0.123	0.000	
**Genetic father**	-0.001 ± 0.240	0.996	0.996	-0.067 ± 0.257	0.794	0.988	-0.154 ± 0.204	0.452	0.956	-0.137 ± 0.200	0.494	0.956
**Genetic mother**	-0.177 ± 0.249	0.477	0. 614	-0.233 ± 0.243	0.338	0. 614	-0.262 ± 0.204	0.200	0. 614	0.054 ± 0.204	0.793	0.892
**Social father**	0.220 ± 0.263	0.403	0.851	0.135 ± 0.306	0.658	0.983	-0.425 ± 0.225	0.059	0.533	-0.057 ± 0.225	0.802	0.983
**Social mother**	0.055 ± 0.279	0.844	0.953	-0.080 ± 0.277	0.774	0. 953	0.057 ± 0.225	0.801	0. 953	0.045 ± 0.230	0.847	0. 953
**LD**	1.121 ± 1.172	0.339	0.422	1.152 ± 1.152	0.317	0.422	1.312 ± 1.153	0.255	0.422	1.215 ± 1.165	0.297	0.422
**LD^2**	-1.339 ± 1.165	0.250	0.310	-1.367 ± 1.141	0.231	0.310	-1.563 ± 1.144	0.172	0.310	-1.429 ± 1.157	0.217	0.310

**Age gen. father**	-0.035 ± 0.207	0.867	0.948	-0.034 ± 0.102	0.737	0.948	0.013 ± 0.204	0.948	0.948	-0.028 ± 0.102	0.787	0.948
**Age gen. mother**	0.136 ± 0.211	0.519	0.685	0.063 ± 0.105	0.548	0.685	0.110 ± 0.211	0.603	0.685	0.053 ± 0.106	0.616	0.685
**Age soc. father**	**0.595 ± 0.242**	**0.014**	**0.026**	**0.287 ± 0.121**	**0.017**	**0.026**	**0.664 ± 0.242**	**0.006**	**0.026**	**0.302 ± 0.124**	**0.014**	**0.026**
**Age soc. mother**	-0.198 ± 0.224	0.377	0.458	-0.099 ± 0.113	0.382	0.458	-0.167 ± 0.225	0.458	0.458	-0.113 ± 0.112	0.314	0.458
**Gen. father x mother**	-0.294 ± 0.567	0.604	0.776	-0.564 ± 0.565	0.318	0.619	0.330 ± 0.402	0.412	0.619	-0.528 ± 0.458	0.249	0.619
**Soc. father x mother**	-0.536 ± 0.610	0.379	1.000	0.428 ± 0.750	0.568	1.000	0.809 ± 0.421	0.055	0.491	0.140 ± 0.455	0.758	1.000

	**MHC-Iα ST9**			**MHC-IIβ DAB1 ST1**			**MHC-IIβ DAB1 ST2**			**MHC-IIβ DAB1 ST5**		
	**Estimate ± SE**	**P value**	**P adj**	**Estimate ± SE**	**P value**	**P adj**	**Estimate ± SE**	**P value**	**P adj**	**Estimate ± SE**	**P value**	**P adj**
**Intercept**	0.680 ± 0.120	0.000		0.692 ± 0.129	0.000		0.705 ± 0.129	0.000		0.690 ± 0.124	0.000	
**Genetic father**	-0.467 ± 0.315	0.139	0.625	-0.491 ± 0.279	0.078	0.235	-0.065 ± 0.202	0.748	0.748	0.636 ± 0.355	0.073	0.235
**Genetic mother**	0.201 ± 0.245	0.412	0. 614	0.260 ± 0.349	0.456	0.522	-0.334 ± 0.206	0.104	0.312	1.069 ± 0.509	0.036	0.215
**Social father**	-0.250 ± 0.349	0.473	0.851	-0.143 ± 0.317	0.652	0.783	-0.001 ± 0.242	0.996	0.996	0.621 ± 0.36	0.085	0.254
**Social mother**	-0.545 ± 0.271	0.044	0. 399	0.675 ± 0.384	0.079	0.420	-0.071 ± 0.248	0.776	0.909	-0.054 ± 0.471	0.909	0.909
**LD**	1.188 ± 1.146	0.300	0.422	1.429 ± 1.232	0.246	0.468	1.463 ± 1.189	0.218	0.468	1.203 ± 1.189	0.312	0.468
**LD^2**	-1.407 ± 1.136	0.216	0.310	-1.686 ± 1.196	0.159	0.339	-1.669 ± 1.175	0.156	0.339	-1.408 ± 1.164	0.226	0.339
**Age gen. father**	-0.146 ± 0.203	0.474	0.948	0.130 ± 0.225	0.563	0.999	0.005 ± 0.204	0.979	0.999	0.143 ± 0.202	0.479	0.999
**Age gen. mother**	0.127 ± 0.207	0.539	0.685	-0.057 ± 0.214	0.789	0.985	0.145 ± 0.207	0.483	0.985	-0.025 ± 0.207	0.903	0.985
**Age soc. father**	**0.505 ± 0.238**	**0.034**	**0.037**	0.414 ± 0.264	0.117	0.117	0.498 ± 0.251	0.048	0.084	0.514 ± 0.243	0.035	0.084
**Age soc. mother**	-0.194 ± 0.219	0.375	0.458	-0.129 ± 0.244	0.597	0.889	-0.112 ± 0.242	0.644	0.889	-0.033 ± 0.237	0.889	0.889
**Gen. father x mother**	-0.166 ± 0.663	0.803	0.903	-0.300 ± 1.278	0.814	0.939	**1.280 ± 0.417**	**0.002**	**0.013**	-1.134 ± 1.286	0.378	0.863
**Soc. father x mother**	-0.013 ± 0.719	0.986	1.000	-	-	-	-0.541 ± 0.463	0.243	0.494	1.232 ± 1.263	0.329	0.494

	**MHC-IIβ DAB1 ST7**			**MHC-IIβ DAB1 ST8**			**MHC-IIβ DAB1 ST11**			**MHC-IIβ DAB2 ST1**		
	**Estimate ± SE**	**P value**	**P adj**	**Estimate ± SE**	**P value**	**P adj**	**Estimate ± SE**	**P value**	**P adj**	**Estimate ± SE**	**P value**	**P adj**
**Intercept**	0.690 ± 0.126	0.000		0.692 ± 0.128	0.000		0.685 ± 0.126	0.000		0.636 ± 0.131	0.000	
**Genetic father**	-0.279 ± 0.333	0.403	0.604	0.161 ± 0.253	0.525	0.630	-0.336 ± 0.215	0.118	0.237	-0.088 ± 0.328	0.789	0.789
**Genetic mother**	-0.265 ± 0.291	0.362	0.522	0.154 ± 0.241	0.523	0. 522	0.218 ± 0.220	0.323	0. 522	0.270 ± 0.372	0.468	0.838
**Social father**	-0.346 ± 0.356	0.331	0.661	0.170 ± 0.293	0.563	0.783	-0.494 ± 0.240	0.040	0.238	**0.751 ± 0.348**	**0.031**	**0.041**

**Social mother**	0.507 ± 0.343	0.140	0.420	-0.292 ± 0.283	0.302	0.603	-0.099 ± 0.252	0.695	0.909	-0.395 ± 0.401	0.325	0.433
**LD**	1.321 ± 1.214	0.277	0.468	0.867 ± 1.214	0.475	0.570	0.515 ± 1.237	0.677	0.677	0.431 ± 1.291	0.739	0.899
**LD^2**	-1.519 ± 1.192	0.203	0.339	-1.063 ± 1.197	0.374	0.449	-0.772 ± 1.219	0.526	0.526	-0.564 ± 1.262	0.655	0.873
**Age gen. father**	0.047 ± 0.203	0.818	0.999	0.000 ± 0.211	0.999	0.999	0.142 ± 0.211	0.500	0.999	0.016 ± 0.225	0.942	0.996
**Age gen. mother**	0.038 ± 0.207	0.855	0.985	0.051 ± 0.206	0.806	0.985	0.004 ± 0.212	0.985	0.985	0.126 ± 0.227	0.580	0.727
**Age soc. father**	0.489 ± 0.251	0.052	0.084	0.482 ± 0.259	0.063	0.084	0.451 ± 0.248	0.070	0.084	**0.674 ± 0.263**	**0.010**	**0.032**
**Age soc. mother**	-0.060 ± 0.239	0.801	0.889	-0.072 ± 0.245	0.767	0.889	-0.037 ± 0.241	0.879	0.889	-0.327 ± 0.245	0.182	0.283
**Gen. father x mother**	-0.085 ± 1.108	0.939	0.939	0.424 ± 0.539	0.432	0.863	-0.189 ± 0.452	0.677	0.939	1.545 ± 0.978	0.114	0.152
**Soc. father x mother**	-0.169 ± 1.149	0.883	1.000	-0.674 ± 0.665	0.311	0.494	-0.601 ± 0.472	0.203	0.494	0.534 ± 0.904	0.555	0.757

	**MHC-IIβ DAB2 ST3**			**MHC-IIβ DAB2 ST8**			**MHC-IIβ DAB2 ST9**		
	**Estimate ± SE**	**P value**	**P adj**	**Estimate ± SE**	**P value**	**P adj**	**Estimate ± SE**	**P value**	**P adj**
**Intercept**	0.652 ± 0.133	0.000		0.658 ± 0.131	0.000		0.609 ± 0.141	0.000	
**Genetic father**	0.125 ± 0.244	0.609	0.789	-0.165 ± 0.306	0.589	0.789	-0.142 ± 0.244	0.560	0.789
**Genetic mother**	-0.029 ± 0.290	0.920	0.920	0.465 ± 0.343	0.176	0.702	0.123 ± 0.255	0.628	0.838
**Social father**	0.025 ± 0.277	0.928	0.928	**-0.762 ± 0.347**	**0.028**	**0.041**	**-0.700 ± 0.257**	**0.006**	**0.026**
**Social mother**	-0.676 ± 0.319	0.034	0.136	0.517 ± 0.369	0.161	0.322	-0.004 ± 0.273	0.989	0.989
**LD**	0.510 ± 1.284	0.691	0.899	0.838 ± 1.264	0.507	0.899	-0.196 ± 1.546	0.899	0.899
**LD^2**	-0.663 ± 1.268	0.601	0.873	-0.994 ± 1.253	0.428	0.873	0.003 ± 1.485	0.998	0.998
**Age gen. father**	-0.020 ± 0.225	0.931	0.996	0.001 ± 0.220	0.996	0.996	0.088 ± 0.237	0.711	0.996
**Age gen. mother**	0.182 ± 0.231	0.432	0.727	0.080 ± 0.228	0.727	0.727	0.113 ± 0.231	0.624	0.727
**Age soc. father**	**0.599 ± 0.265**	**0.024**	**0.032**	**0.532 ± 0.255**	**0.037**	**0.037**	**0.616 ± 0.270**	**0.023**	**0.032**
**Age soc. mother**	-0.346 ± 0.251	0.167	0.283	-0.280 ± 0.245	0.253	0.283	-0.266 ± 0.248	0.283	0.283
**Gen. father x mother**	0.396 ± 0.638	0.535	0.535	1.529 ± 0.951	0.108	0.152	-0.797 ± 0.500	0.111	0.152
**Soc. father x mother**	0.228 ± 0.668	0.733	0.757	-0.285 ± 0.922	0.757	0.757	-0.474 ± 0.520	0.361	0.757

Table S17. Post-hoc Tukey pairwise comparisons of the fledging success between all **genetic** couples regarding the presence or absence of MHC-IIβ DAB1 supertype 2 in each parent. “0”: absence, “1”: presence; “G♂”: genetic father, “G♀”: genetic female. Tukey adjustment for multiple comparisons was applied and tests were performed on the *log* odds ratio. Results are averaged over the other covariates.

Contrasts					Odds ratio	SE	*z* ratio	P value
**G♂**	**G♀**	**–**	**G♂**	**G♀**				
0	0	–	1	0	2.308	0.767	2.516	0.057
**0**	**0**	**–**	**0**	**1**	**2.618**	**0.767**	**3.285**	**0.006**
0	0	–	1	1	1.680	0.507	1.719	0.314
1	0	–	0	1	1.134	0.322	0.444	0.971
1	0	–	1	1	0.728	0.213	-1.083	0.700
0	1	–	1	1	0.642	0.162	-1.752	0.297
